# Mood fluctuations shift cost–benefit tradeoffs in economic decisions

**DOI:** 10.1038/s41598-023-45217-w

**Published:** 2023-10-24

**Authors:** Roeland Heerema, Pablo Carrillo, Jean Daunizeau, Fabien Vinckier, Mathias Pessiglione

**Affiliations:** 1 Motivation, Brain and Behavior (MBB) Lab, Paris Brain Institute (ICM), Pitié-Salpêtrière Hospital, 75013 Paris, France; 2 Sorbonne Université, Inserm U1127, CNRS U7225, 75013 Paris, France; 3 Université Paris Cité, 75006 Paris, France; 4 Department of Psychiatry, Service Hospitalo-Universitaire, GHU Paris Psychiatrie and Neurosciences, 75014 Paris, France

**Keywords:** Human behaviour, Computational neuroscience, Emotion, Motivation

## Abstract

Mood effects on economic choice seem blatantly irrational, but might rise from mechanisms adapted to natural environments. We have proposed a theory in which mood helps adapting the behaviour to statistical dependencies in the environment, by biasing the expected value of foraging actions (which involve taking risk, spending time and making effort to get more reward). Here, we tested the existence of this mechanism, using an established mood induction paradigm combined with independent economic choices that opposed small but uncostly rewards to larger but costly rewards (involving either risk, delay or effort). To maximise the sensitivity to mood fluctuations, we developed an algorithm ensuring that choice options were continuously adjusted to subjective indifference points. In 102 participants tested twice, we found that during episodes of positive mood (relative to negative mood), choices were biased towards better rewarded but costly options, irrespective of the cost type. Computational modelling confirmed that the incidental mood effect was best explained by a bias added to the expected value of costly options, prior to decision making. This bias is therefore automatically applied even in artificial environments where it is not adaptive, allowing mood to spill over many sorts of decisions and generate irrational behaviours.

## Introduction

Mood is typically construed as an affective state that chiefly fluctuates on a valence dimension (between happiness and sadness), that is not precisely related to one specific trigger, that can last for some time, and incidentally exert pervasive effects on thoughts and actions. From the perspective of rational decision theory, mood fluctuations are viewed as undesirable, because they drive us to make poor decisions. Indeed, we often miss interesting opportunities in moments of sadness, and embrace foolish enterprises in moments of happiness. Decision biases induced by incidental mood changes have been documented by statistical observations of real-life behaviours. Classical examples are that people are more inclined to buy a lottery ticket, or to invest in some stock market, on days when the weather is nice, or after the victory of their favourite sports team^1–5^. These decision biases have been reproduced at a shorter time scale in the laboratory, using newspaper reports or small gifts to induce mood changes^6–8^. Although comparisons between groups using a single mood-inducing stimulus yielded mixed evidence^9–11^, comparisons between episodes created with different inductions showed that participants were generally more prone to take risks when in a good mood, and avoid risks when in a bad mood^12–15^. These effects qualify as decision biases because choice outcomes are independent from the events that triggered mood changes (e.g., lottery outcomes are independent from sport events). To be rational in this perspective, a decision-maker should coldly consider what relates to the attributes of choice options, and nothing else.

However, an opposite view has emerged, building on the idea that, if mood flexibility has been favoured by natural selection, it must provide an adaptive advantage^16,17^. By flexibility we mean here the ability to vary as a function of life events. A key suggestion is that mood may provide a valid generalization of reward estimates over time and actions^18^. The generalization is indeed valid in environments where sources of reward are both auto-correlated (across time) and inter-correlated (between them). This is typically the case with seasonal variations for hunter-gatherers: the appearance of accessible fruits at the beginning of spring is associated with more of these fruits getting ripe over the next weeks, together with other types of fruits, and also with little preys coming out their nests and burrows. If gathering fruits improve mood, and if good mood favours foraging behaviour, then rather than driving irrational decisions, mood fluctuations would actually help adjusting the behaviour to the statistics of the environment.

In the initial formalization of this seminal intuition^19^, mood was assumed to accelerate learning of reward availability by amplifying reward prediction errors associated with action outcomes. Although interesting, this mechanism misses the fact that mood can have a direct impact on decisions, by shifting the values of choice options, without resorting to learning processes that would only update values when outcomes are observed^20,21^. This direct decision bias has been modelled in the case of choice under risk, and associated with the activity of brain regions that implement the valuation of positive and negative prospects^12,15^. In a recent theoretical paper^22^, we proposed that shifting attitude towards risk is just one case of the general impact that mood may have on cost–benefit tradeoffs. Indeed, other types of cost, such as time and effort, may be as important as risk in the decisions to forage or not for particular rewards. Therefore, mood fluctuations should impact how rewards are discounted, not just by risk, but also by time and effort. Moreover, if the bias is automatically triggered, mood effects on decisions should be observed even when not adaptive, that is even in situations where mood triggers and choice options are artificially decorrelated.

To test this assumption, we designed a new behavioural task that combines the same mood induction procedure, using quiz questions and feedbacks as in previous papers^12,15^, with economic choices in which monetary rewards are traded against different types of cost (risk, delay, physical effort, mental effort). Choices always oppose a small amount of money at no cost to a bigger amount at a higher cost, as is classic in the neuroeconomic literature^15^. The same kind of choices have already been implemented in a previous study that explored the effects of cognitive fatigue on economic decisions^23^. The aim here was thus to systematically assess the effects of high and low mood induction on how the different types of costs are traded against the same monetary rewards. Consistent with our theory, we observed that high/low mood biased all choices toward accepting/declining to incur higher costs for bigger rewards, irrespective of which type of cost was associated with the reward. Computational modelling of choice behaviour indicated that mood effects were best explained by an additive shift of decision values favouring either the costly or uncostly options.

## Results

Hereafter, we describe an experiment featuring a mood induction procedure interleaved with an economic choice task. This experiment, conducted on 102 participants (76 females/26 males, mean age = 32.5 ± 1.6), is referred to as the main study, by opposition to pilot studies 1 and 2, respectively conducted on 25 and 21 participants, and described in the Supplementary Information (with demographic details in Table S1).

### Mood fluctuations

By design, the tasks meant to induce mood fluctuations (with feedbacks on quiz questions) were independent from the tasks meant to reveal their effects (on economic decisions).

**Figure 1:**
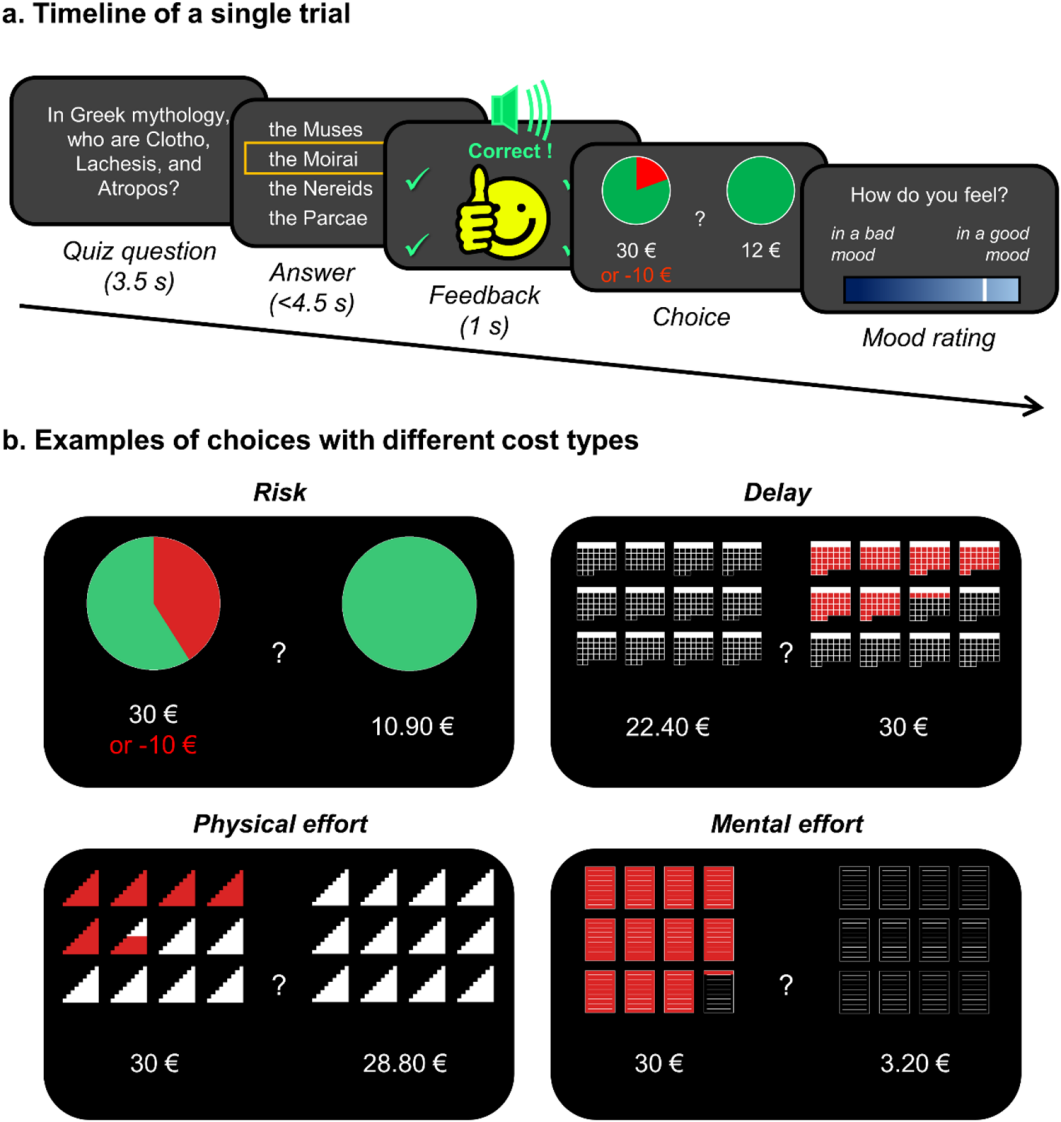
Behavioural tasks. (**a**) Timeline of a single trial example. A quiz question was presented for 3.5 s, followed by a screen with four answer options. On a tactile screen, participants had to select an answer within 4.5 s and immediately received visual and auditory feedback lasting for 1 s. Then they made one economic decision by selecting which of two options they preferred. Finally, they rated their mood by positioning a cursor on a visual analogue scale. Economic choice and mood rating were self-paced. (**b**) Choice examples probing how monetary rewards are discounted with the different types of costs (risk, delay, physical effort, mental effort). The two options were either a variable small reward at no cost, or a 30€ reward obtainable at a variable cost. The costs in the examples shown entailed a 42% risk of losing 10€ instead of getting the reward (risk discounting), a delay of 9 months and 1 week until reception of the reward (delay discounting), climbing 5 flights of stairs and 6 steps (physical effort discounting), and copying 11 pages plus 1 line of text in a foreign language (mental effort discounting).

**Figure 2:**
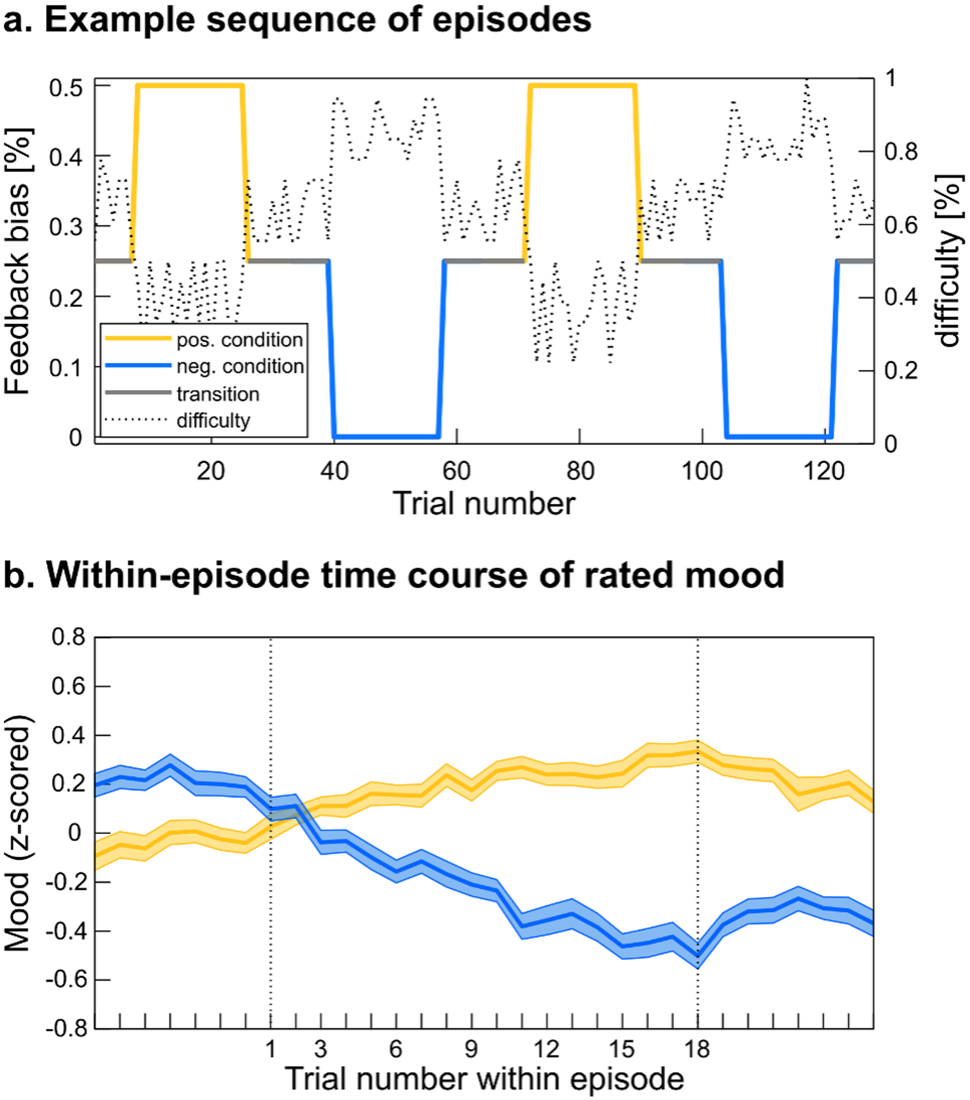
Mood fluctuations. (**a**) One experimental session of an example participant. The coloured solid line visualizes the feedback bias (proportion of incorrect answers given a positive feedback): 50% in positive episodes (yellow), 25% in the transition between episodes (grey), 0% in negative episodes (blue). In addition, the dotted line displays the a priori difficulty (rate of incorrect answers obtained from an independent group) of each presented question. Questions were sorted and split in three levels of difficulty assigned to positive episodes, transitions, and negative episodes. (**b**) Effect of feedback on mood (z-scored rating). The plot show an average over trials surrounding positive and negative episodes, with no baseline correction. Solid lines show the rated mood averaged across participants, shaded areas represent inter-participant standard errors around the mean.

To evoke positive and negative mood episodes, we adopted a previously established paradigm^15^ that has since been replicated^12^. Participants played a general-knowledge quiz featuring multiple-choice questions. Feedback was given immediately upon answering a quiz question, after which participants first made an economic decision and then rated their current mood. Completing one trial thus consisted in answering one quiz question, making one economic choice, and providing one mood rating (Fig. 1a). The order of rating and choice was reversed in comparison to our previous design, with the aim to avoid a direct effect of expressing mood on making decisions. Mood was manipulated through the positive/negative feedbacks, which consisted of a happy/sad emoji and a bell/buzz sound. Correct answers were always followed by positive feedbacks. Critically, feedbacks given to incorrect answers depended on an experimentally controlled bias that varied across conditions. There were 3 conditions, with a 50% bias during positive episodes, a 0% bias during negative episodes, and a 25% bias for transitions between episodes. The bias here indicates the proportion of incorrect answers that are given positive feedback. Because participants were actually wrong on most quiz questions, the bias manipulation created sequences with high, medium and low rate of positive feedbacks (Fig. 2a). As an additional manipulation, questions asked during positive and negative episodes were respectively easier and harder, while during transitions between episodes they were intermediately difficult. The question difficulty was determined before the experiment as the rate of incorrect answers made by a different group of participants in a previous study. This second manipulation amplified the difference in positive feedback rate between episodes, and thereby the amplitude of mood fluctuations.

The main study comprised two sessions, scheduled approximately two weeks apart. This was done in the interest of a larger project assessing the replicability of behavioural tests, which is beyond the scope of this paper. An experimental session comprised two positive and two negative episodes in a random order. Each episode spanned 18 trials and was preceded and followed by 7 transition trials. Here we report results from both sessions pooled together (for a total of 2 × 128 = 256 trials).

Reflecting the manipulation of question difficulty, the mean correct-response rate was 21.6%, 35.8% and 50.6% during negative episodes, transitions, and positive episodes, respectively. With the additional bias manipulation, the positive feedback rate was respectively 21.6%, 52.1% and 76.2%. Consistently, mood rating increased / decreased in the course of positive / negative episodes and returned to baseline between episodes (Fig. 2b). Note that mood was gauged at every trial in the present protocol, such that no interpolation was needed, contrary to previous studies^12,15^.

We tested the success of the mood manipulation, separately for the two sessions, by testing the difference between mood ratings given during positive vs. negative episodes. During both sessions, mean rated mood was significantly higher in positive than in negative episodes (*Δ*_*session 1*_ = 0.45 ± 0.07, *Δ*_*session 2*_ = 0.35 ± 0.07; *t*_*1*_(101) = 6.41, *t*_*2*_(101) = 5.25; *p*_*1*_ = 4.5E−9, *p*_*2*_ = 8.4E−7), with no significant difference between the two (*t*(101) = 1.341, *p* = 0.183). Thus, repeating the same experiment with different questions two weeks apart did not affect the success of mood induction, allowing the two sessions to be pooled together. We also tested the success of positive and negative inductions separately, by testing against zero the slope of mood ratings calculated across trials of a same episode. Both positive and negative inductions were significant (*β*_*pos*_ = 0.015 ± 0.003 and *β*_*neg*_ = − 0.035 ± 0.004, *t*_*pos*_(101) = 5.54 and *t*_*neg*_(101) =  − 9.09, *p*_*pos*_ = 2.43e−7 and *p*_*neg*_ = 9.66e−15). Negative induction was significantly more efficient than positive induction (*t*(101) = 4.63, *p* = 1.12e−5), an asymmetry that was already observed in previous studies^12,15,18^. Finally, we tested whether the efficiency of mood induction would decline with the repetition of episodes, by comparing the first and second halves of sessions (pooling the two sessions). The difference in mood rating slopes between positive and negative episodes was significantly different from zero in both halves (Δ*β*_early_ = 0.054, Δ*β*_late_ = 0.045, both *t*(94) > 6.48, both *p* < 4.2e−9), with no difference between the two (*t*(94) = 1.06, *p* = 0.292), arguing against a possible decline in the efficiency of our mood induction procedure.

Thus, the design of the main study proved to induce robust mood fluctuations. In pilot studies 1 and 2, we have tried variants around the same methodology (see Table S2 for a comparison of the three protocols). Of note, we used 4 different scales (‘happy’, ‘sad’, ‘calm’, ‘tense’) in study 1, with the aim to decompose mood into independent sub-dimensions, such as valence (happy versus sad) versus arousal (calm versus tense). However, these supplementary scales did not provide much additional information, as participants reported feeling both happy and calm in positive episodes, and both sad and tense in negative episodes. Indeed, happy minus sad rating was strongly correlated to calm minus tense rating (*ρ* = 0.40 ± 0.07, *t*(24) = 6.12, *p* = 3E−6). Also, we intended to implement more gradual transitions between positive and negative moods, with neutral episodes giving no feedback (study 1) or additional levels of feedback bias (study 2). Again, these variants did not seem to augment the amplitude of mood fluctuations, so we did not retain them for the main study. Nevertheless, in both pilot studies the mean rated mood significantly diverged when comparing positive and negative episodes (Fig. S1).

### Choice behaviour

At the end of a trial, participants made an economic decision between a small reward delivered with no cost and a bigger reward obtainable at some cost (Fig. 1b). The big reward was always 30€ but the small reward varied across trials (between 0.1€ and 29.9€). The cost type and level also varied across trials. There were 4 possible types: a risk of losing money, a delay to payment, a physical effort (climbing flights of stairs), or a mental effort (copying a cryptic text). The two variables (small reward and cost level) were selected using an online trial generation (OTG) algorithm (see “Methods”).

**Figure 3:**
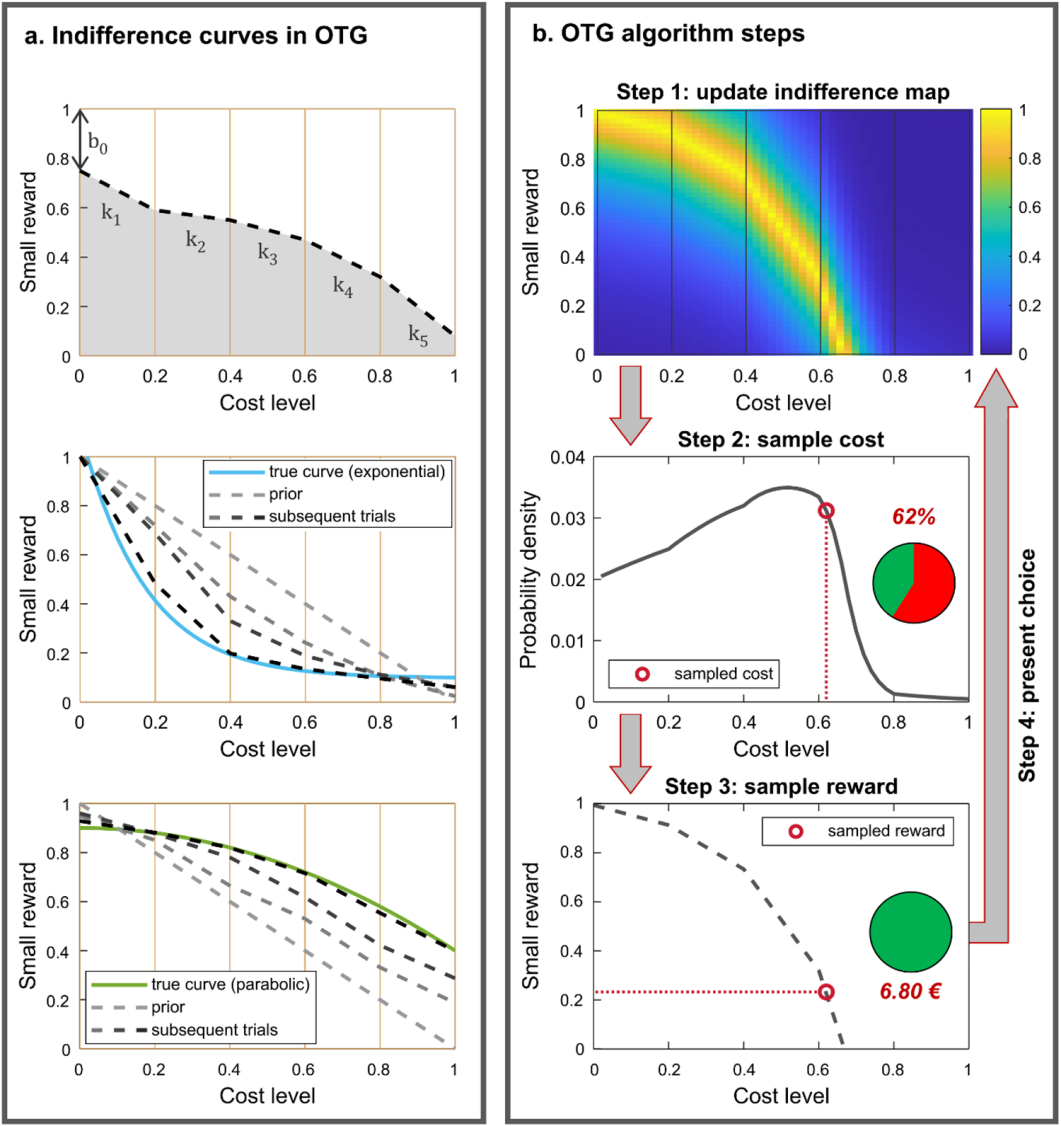
Online trial generation. (**a**) Modelling the indifference curve. The effect of mood is presumably maximal in decisions between options of similar values, for which the participant has no strong preference. To generate such decisions, it is necessary to identify indifference points within a 2D space formed by the two variables that varied across trials: the small reward and the cost associated with the big reward. To avoid commiting to any discounting shape, we modelled this indifference curve as 5 edge-constrained linear functions fitted to the 5 adjacent cost bins (top panel). This piecewise linear function therefore included 6 parameters in total: the intercept and the 5 slopes. When adjusted over successive trials (light to dark gray dashed lines) to best describe the observed choices, the piecewise linear mapping can approximate any discounting shape, as for instance a convex exponential-decay function (middle panel), or a concave parabolic-decay function (bottom panel). (**b**) Generating choice options. After every trial, a map of the indifference space is computed given the updated estimates of the 6 parameters (step 1). Brighter colours in the top panel correspond to (reward, cost) combinations for which choice probability is closer to 50%. From this map, a probability density function is derived, under which a cost level is randomly drawn (step 2). Then, the small reward that is expected to yield an indifferent choice for this particular cost level is identified (step 3). The selected cost level and small reward are finally presented to the participant at the next choice trial (step 4).

We reasoned that if moods influence decisions, this is more likely to manifest in choices for which the participant has no strong prior preference between the two options. To maximize the sensitivity of our choice trials, we therefore focused on choice options for which the participant is close to indifference. The issue is that different participants may have different preferences regarding how reward should be discounted with risk, delay or effort. To account for these subjective preferences, it is common practice to use discount functions with free parameters fitted to the participant’s choices. When these preferences need to be identified for the generation of test trials, as in our case, the parameters are typically fitted to choices made during a pre-test calibration phase of the experiment. Because this calibration procedure takes time and may by itself influence mood, we opted for a procedure that updates indifference curves continuously throughout the experiment. A key advantage of such an OTG procedure is keeping track of indifference points, and hence presenting sensitive choices, even if preferences vary with mood induction or drift with time on task. A drawback, however, is that the procedure mechanically goes against any effect of the mood manipulation, by proposing better costly options if they are not chosen, or better uncostly options in the opposite case. Another inconvenience is that the choice model must be defined before observing the choices. If it so happens that the model is unable to account for the participant’s choices, then all trials will present options that are off the indifference curve. To mitigate that concern, we opted for an agnostic parameterization of the indifference curve, in which the value of rewards decays linearly within pre-specified cost intervals. The advantage of this piecewise linear function is that it can approximate any discounting shape, whether convex or concave (Fig. 3a). The piecewise linear function was updated after every choice and used to draw a reward/cost combination under a probability distribution that scaled with the estimated likelihood of indifference (Fig. 3b). This eventually focuses the distribution of options presented across trials around indifference points.

To compare the performance of the OTG procedure with other sampling procedures, we ran simulations of dummy choice trials based on various discount functions (see “Methods”). Two extreme approaches to sampling the reward-cost space are random sampling (based on a uniform probability distribution) and grid sampling (at regular intervals). When compared to these approaches (Fig. S3), the OTG algorithm better predicted the simulated choices (with a higher balanced accuracy) and provided better estimates of the model parameters (with smaller posterior variance). We also checked that the reward and cost levels selected by the OTG algorithm were independent from mood fluctuations. Indeed we found no difference between positive and negative episodes, neither in mean reward magnitude (*μ*_*pos*_ = 54.5 ± 1.1%, *μ*_*neg*_ = 55.5 ± 1.2%, *t*(101) = 1.47, *p* = 0.146), nor in any of the 4 mean cost levels (41.2 ± 1.1% < *μ*_*pos*_ < 51.2 ± 0.9%, 41.5 ± 1.2% < *μ*_*neg*_ < 50.2 ± 0.8%, 0.092 < *t*(101) < 1.03, 0.268 < *p* < 0.927).

**Figure 4:**
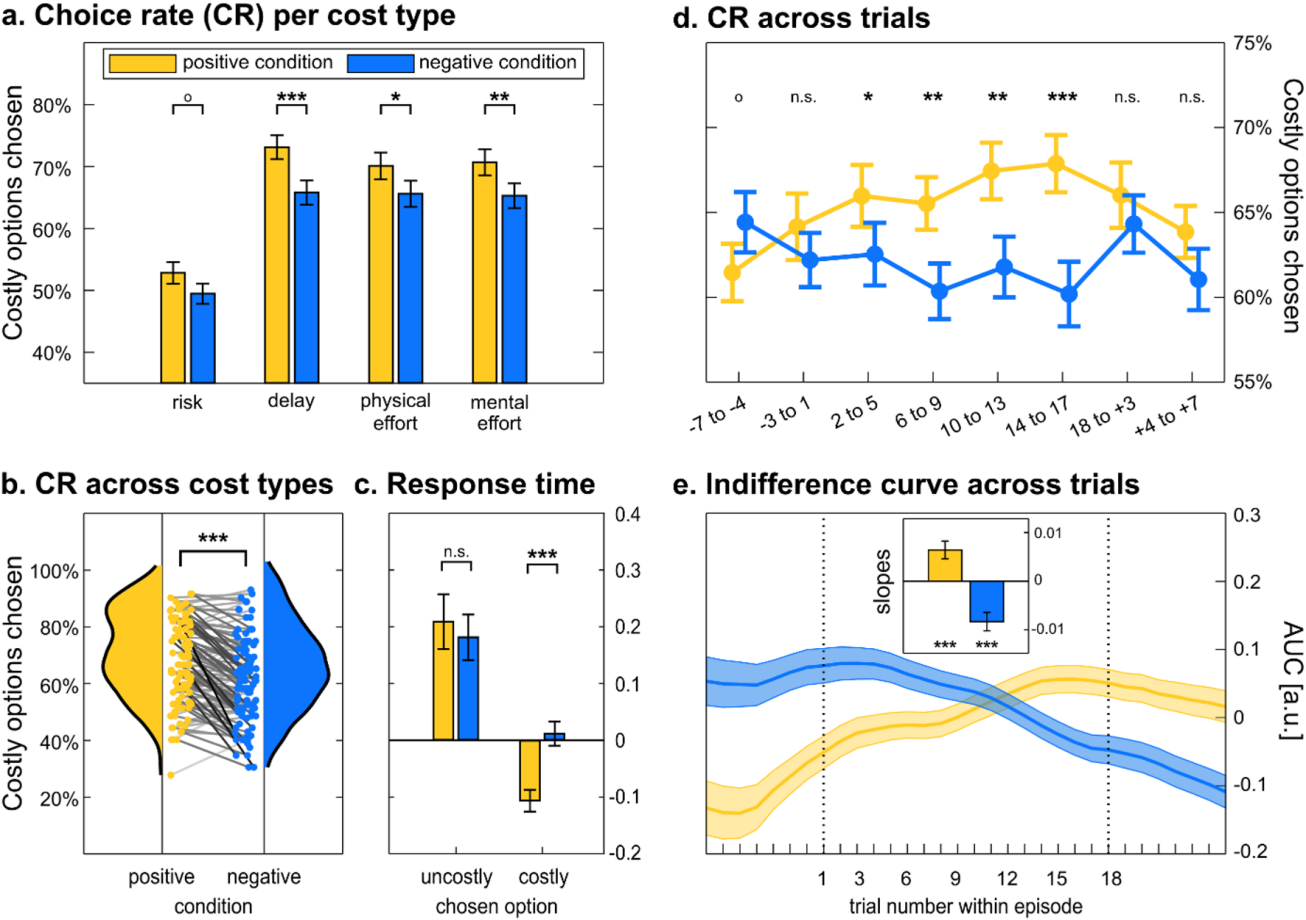
Effects of mood induction on choice behaviour. (**a**) Comparison of costly choice rates, shown per cost type, between positive (yellow) and negative (blue) episodes. (**b**) Comparison of costly choice rates across all cost types (figure generated with the Raincloud plot toolbox, ref.^25^). Dots are individual participants included in the global distributions. Shades of grey indicate per-participant effect size (difference between positive and negative episodes). (**c**) Comparison of z-scored choice response time (RT) between positive and negative episodes. (**d**) Time course of costly choice rates, across cost types, around positive and negative episodes. Choices were binned per series of four consecutive trials that probed the four cost types. (**e**) Time course of the area under the curve (AUC), across cost types, around positive and negative episodes. The inset shows the mean AUC slopes over all trials within an episode. In all panels, error bars are inter-participant standard errors of the mean. *p < 0.05, **p < 0.01, ***p < 0.001, °p < 0.1, *n.s.* not significant.

As a model-free assessment of mood effects, we simply compared the choices made during positive and negative episodes. Across the different types of cost (Fig. 4a), there was a significant difference of 5.1 ± 1.1% (*t*(101) = 4.76, *p* = 6.4E−6), meaning that participants were taking the costly option more often during positive than during negative episodes. This effect was again significant in both sessions (*Δ*_*session 1*_ = 5.24 ± 1.5%, *Δ*_*session 2*_ = 5.06 ± 1.4%; *t*_*1*_(101) = 3.50, *t*_*2*_(101) = 3.59; *p*_*1*_ = 7E−4, *p*_*2*_ = 5E−4), with no significant difference between the two (*t*(101) = 1.341, *p* = 0.183), which were therefore analysed together. Also, the effect was significant in both the first and second halves of sessions (*Δ*_*early*_ = 5.23 ± 1.5%, *Δ*_*late*_ = 5.05 ± 1.4%; *t*_*1*_(101) = 3.50, *t*_*2*_(101) = 3.58; *p*_*1*_ = 7E−4, *p*_*2*_ = 5E−4), with no significant difference between the two (*t*(101) =  − 0.09, *p* = 0.926), arguing against an attenuation of mood influence on decisions. When looking at the four cost types separately (Fig. 4b), the difference was in a similar range (from 3.4 ± 1.8% to 7.3 ± 1.6%) and was either significant or bordering significance (from *p* = 1.8E−5 to *p* = 0.064). We then investigated whether the difference in choice rate was correlated across participants between cost types taken two by two. All correlations were significant when only considering delay, physical effort, and mental effort (all *ρ* > 0.42, all *p* < 1E−5), but the difference observed for risk was unrelated to the other cost types (all |R|< 0.08, all *p* > 0.45). However, a direct comparison showed no significant effect of cost type (one-way ANOVA: *F*(3,404) = 0.98, *p* = 0.403), hence no reason to single out choice under risk, except for the difference in baseline choice rates.

We then inspected the time course of preference shift across trials within an episode. Choice rate closely followed the dynamics of mood rating (compare Fig. 4d and Fig. 2b), with the difference between positive and negative conditions emerging in the first trials and peaking in the last trials of an episode. Even if it revealed significant mood effects, choice as a measure has limitations, first because it is binary and second because it is counteracted by the OTG procedure. To get a more sensitive measure, we turned to the area under the indifference curve that is systematically updated through the OTG procedure (Fig. 4b). This time-resolved continuous measure is a proxy for the global willingness to accept the costly option. Critically, this measure increased across trials during positive episodes (*β*_*pos*_ = 0.006, *t*_*pos*_(101) = 3.54, *p* = 6E−4), and decreased during negative episodes (*β*_*neg*_ = − 0.008, *t*_*neg*_(101) =  − 4.38, *p*_*corr*_ = 3E−5). There is a lag, however, between the time courses of AUC and choice rate (compare Fig. 4d and e), due to the facts that the AUC can only be updated once a choice is made, and that each cost type was only probed every four trials.

Next, we examined choice response time (RT), which is known to decrease when the decision value increases [e.g.,^24^]. Because in our theory mood augments decision value (toward high-reward high-cost options), we expected an interaction between mood and choice (i.e., participants should be faster to choose a costly option when in a positive mood). We regressed RT against a linear model including the main effects and interaction of the two factors: mood level (positive versus negative episode) and observed choice (costly versus uncostly option). The interaction was indeed significant (*β*_*Choice* × *Condition*_ = 0.14, *t*(101) = 3.24, *p* = 0.002), indicating that participants were globally faster at choosing costly options and even more so when in a good mood (Fig. 4c).

Thus far, we have compared episodes corresponding to the positive and negative moods induced by the protocol. To better link choices to the actual mood experienced by participants, we ran a logistic regression of choice against mood rating (given in the same trial). We found that indeed, rated mood positively predicts costly decisions (*β*_*mood*_ = 0.10, *t*(101) = 4.05, *p* = 1E−4), an effect that was present in both sessions (*β*_*session 1*_ = 0.028, *β*_*session 2*_ = 0.028; *t*_*1*_(101) = 2.48, *t*_*2*_(101) = 3.21; *p*_*1*_ = 0.015, *p*_*2*_ = 0.002), with no significant difference between the two. To test whether this effect was only driven by the last feedback or by a mood construct that would integrate previous feedback, we ran a multivariate logistic regression of choice against the feedback received in the previous trial as a categorical regressor (positive or negative), and the mood rating given in the previous trial as a continuous regressor. We found that the feedback just received has the largest impact on the willingness to take the costly option (*β*_*Feedback*_ = 0.31, *t*(101) = 6.45, *p* = 4E−9), but that mood rated before this last feedback also had a significant impact (*β*_*Previous Mood*_ = 0.07, t(101) = 3.06, *p* = 0.003).

To better disentangle the mood effect from a direct effect of feedback, we conducted a regression across participants. For each participant, we calculated the mean difference in positive feedback rate, mood rating and choice rate, between positive and negative episodes. Then the differences in feedback rate and mood rating were both included in a regression model meant to explain the difference in choice rate. We found that the difference in choice rate was significantly predicted by the difference in rated mood (*β*_*Δ mood*_ = 0.04 ± 0.02, *t* = 2.26, *p* = 0.026) but not by the difference in feedback rate (*β*_*Δfeedback*_ = − 0.17 ± 0.13, *t* = − 1.27, *p* = 0.205).

Thus, the mood effects in the main study were strong and robust, as they generalized across cost types and remained unchanged when retested two weeks later. However, the design of the main study benefited from two pilot studies in which mood effects were less clear (Fig. S2). The impact of mood on costly choice was not significant in pilot study 1, where mood was reconstructed post-hoc from separate ratings of happiness and sadness. In pilot study 2, the effect of rated mood on costly choice was of the same size as in the main study, but statistically weaker due to the small sample (*β*_*mood*_ = 0.116, *t*(20) = 2.58, *p* = 0.018). This result nonetheless shows that the same mood effect on choice can be obtained even when choices are incentivized (as they were in pilot study 2). Also, this study enabled testing the order between rating and choice: there was no significant difference in mood rating, whether it was given before or after the choice (Δ_before_ = 0.42, Δ_after_ = 0.31, *t*(20) = 1.13, *p* = 0.274), arguing against the possibility that the effect on mood was contaminated by the effect on choice. Regarding the effect on choice, it is difficult to formally assign the improvement over studies to a specific factor, because we made many changes in the design (see Tables S2 and S3 for a comparison). A key improvement after pilot study 1 might be the adoption of the OTG procedure to make choices more sensitive to mood fluctuations.

### Model-based analyses

Following the above model-free results, we aimed to disentangle the contribution of mood from that of option attributes (reward and cost levels). For this we used computational modelling of choice behaviour, to account for mood effect with parameter estimation, an approach that is independent from the OTG procedure (contrary to AUC and choice rate). Note that the OTG procedure, which was performed during the experiment prior to the model-based analyses, did not use any discount function but a piecewise linear approximation that could be flexibly adjusted to any form of discounting. Our computational approach was to first identify the model that best fits choices, irrespective of mood, and then to complement that model with a rated mood factor weighted by an additional parameter. The model includes a discount function that generates subjective values and a softmax function that maps value differences onto choice probabilities.

We tested different discount functions for the different types of cost, as previous studies reported systematic differences. For example, both theoretical analysis and empirical evidence^23^ suggest that delay discounting is best captured by a multiplicative convex function that asymptotically decays to zero, whereas effort discounting might rather follow an additive concave function that can produce negative discounted values. We thus started with basic functions commonly used in the literature, with one weight parameter (a discount factor) for each type of cost. Then we tested variants including additional parameters for the weight on reward and power on costs (to control the curvature of the discount function). Regarding the softmax function that provides a sigmoid mapping from decision value (difference between options) to choice probability, we also tested variants with different inverse temperature and additive bias parameters for the different types of costs. The resulting 36 models (see “Methods” for a full description) were inverted and compared using the Variational Bayesian Analysis toolbox^26^. The most plausible model (with 94% exceedance probability), according to Bayesian model comparison (see full results in Table S4) comprised the following discount functions:

1 $${V}_{R}={k}_{Rew}\cdot Rew\cdot P-{k}_{R}\cdot L\cdot \left(1-P\right),$$

where $P$ is the probability of winning and $L$ the amount that can be lost.

2 $${V}_{D}={k}_{Rew}\cdot Rew\cdot \mathit{exp}\left(-{k}_{D}\cdot D\right),$$

where $D$ is the delay.

3 $${V}_{PE}={k}_{Rew}\cdot Rew-{k}_{PE}\cdot {PE}^{2},$$

where $PE$ is the physical effort.

4 $${V}_{ME}={k}_{Rew}\cdot Rew-{k}_{ME}\cdot {ME}^{2},$$

where $ME$ is the mental effort.

**Figure 5:**
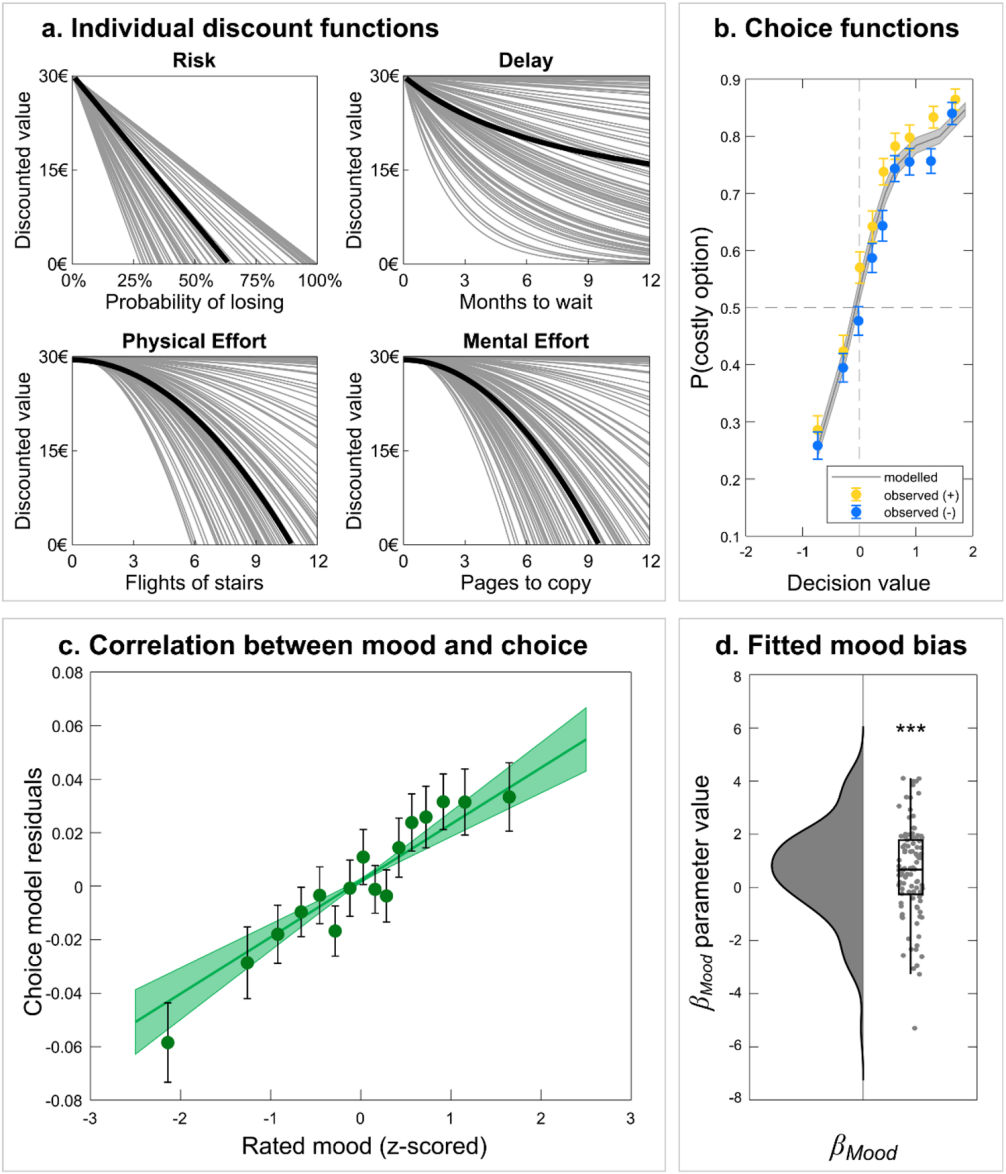
Model-based analysis of mood effects on choice behaviour. (**a**) Individual discount functions for the different cost types. Discount factors were obtained by fitting the model to all choice trials. For illustrative purposes, reward weight parameters have been fixed to 1, such that all individual discount functions start at 30€ (corresponding to the large reward offered for all costly options). The black lines show the median discount functions over participants. (**b**) Observed and modelled choice functions. Dots show the observed probability of choosing the costly option, separately for positive (yellow) and negative (blue) episodes, as a function of decision value bins. Decision value (x-axis) and choice probability (grey line) were computed using the winning model with no mood effect. Error bars and shaded areas are inter-participant standard errors of the mean. (**c**) Correlation between choice residuals and rated mood. Dots show residuals from the choice model fit, grouped in 16 bins according to z-scored rated mood, with linear regression fit averaged across participants. Error bars and the shaded area denote inter-participant standard errors of the mean. (**d**) Individual mood bias parameters and overall distribution. A positive parameter indicates an increased tendency to choose the costly option along with improved mood, and vice versa for the uncostly option as mood deteriorates. Figure generated with the Raincloud plot toolbox^25^. ***p < 0.001.

Here, the reward weight ${k}_{Rew}$ is common to all cost types, while *k*_*R*_, *k*_*D*_, *k*_*PE*_, and *k*_*ME*_ are specific weight parameters for risk, delay, physical effort, and mental effort, respectively. Specific weight parameters must be used for costs because the risk, delay and effort levels are expressed in different units, so they must be scaled to account for how they compare to the reward unit, which was 1€ in all cases, allowing for a single weight parameter. All fitted discounted functions are illustrated in Fig. 5a. Note that risk and effort discounting functions can generate negative values, whereas values discounted by delay are always positive. Intuitively, this conforms with the notion that making an effort or taking the risk of losing money can be worse than doing nothing (no cost, no reward), whereas obtaining a reward after a delay is always better than nothing.

The softmax function can be written as a sigmoid transformation of the decision value *DV*, with *P(costly)* denoting the probability of selecting the costly option:

5 $$P\left(costly\right)=\frac{1}{1+exp(-{\beta }_{0}- {\beta }_{1}\cdot DV)},$$

where *DV* is *V(costly) − V(uncostly).*

In the winning model, both the inverse choice temperature *β*_*1*_, which controls the consistency of choices, and the choice bias *β*_*0*_, which controls the willingness to take the cost (irrespective of option values), were specific to each cost type.

When plotting choices as a function of decision value, we observed a vertical shift between positive and negative episodes, with the modelled choices averaged over all trials falling in between (Fig. 5b). This suggests that mood modulates the additive bias parameter to shifts choice probability upwards, irrespective of option values. Accordingly, rated mood was significantly correlated (*R* = 0.052 ± 0.01, *t*(101) = 4.57, *p* = 1.4E−5) with choice residuals, after explaining away the effects of option attributes with the fitted model (Fig. 5c). We therefore included rated mood (i.e., the mood rating given in the same trial as the choice made by the participant) as a modulator of the additive bias in the softmax function:

6 $$P\left(costly\right)=\frac{1}{1+exp(-{\beta }_{0}\cdot (1+{\beta }_{Mood}\cdot Mood)- {\beta }_{1}\cdot DV)}$$

The parameter *β*_*Mood*_, which allows for a modulation of the choice bias by the rated mood, was shared across all cost types. Before fitting this model, we ran simulations to verify that the *β*_*Mood*_ parameter is recoverable. We observed that indeed the correlation between fitted and simulated parameters is close to 1, particularly when using OTG rather than other sampling methods (Fig. S3). After fitting this augmented model to all observed choices (Fig. 5d), we tested the posterior estimates of the mood weight parameter which were significantly positive at the group level (*β*_*Mood*_ = 0.614, *t*(101) = 3.54, *p* = 6.1E−4). Finally, we compared this model to an alternative model in which mood is not affecting the additive choice bias but the multiplicative weight on reward. Bayesian model comparison indicated that the additive model is by far more plausible than the multiplicative model (with an exceedance probability of 99.9%). Note that an alternative multiplicative model in which mood would affect the weights on costs would need four parameters and hence would not stand a chance against the additive model. Thus, model-based analyses concurred to show that choices are biased toward costly options when participants experience a higher mood, and towards uncostly options when in a lower mood. This additive bias is independent from option attributes, as if representing a prior preference for facing costs and obtaining more rewards, whatever these costs and rewards might be.

## Discussion

In this study, we have systematically tested the effects of induced mood fluctuations on economic choices involving tradeoffs between monetary rewards and different types of costs. The induction procedure was validated by subjective ratings demonstrating episodes of high and low moods. Whatever the cost type (risk, delay, physical effort, mental effort), preferences were shifted toward more rewarded but costly options, during positive relative to negative mood episodes. In computational analyses, this effect was best explained by a bonus proportional to self-reported mood that was added to the costly option value, prior to making a decision.

To our knowledge, the different types of costs had never been gathered in a same study exploring behavioural effects of mood fluctuations. However, these costs had been studied separately, including risk attitude, delayed gratification and effort expenditure, with contrasted results (e.g.,^7–9,26–31^). The effects observed here were consistent across cost types, highly significant and robust to test–retest in successive visits. Baseline choice rates were different with risk relative to the other costs, but this likely relates to a difference in the tailoring of choices around indifference points. A conceptual difference is that, unlike with the other costs, there is a normative way to integrate risk with reward (by multiplying probability and magnitude), so participants might have engaged in an explicit calculation of expected values for risky options. Despite these empirical and conceptual differences, mood effects on risky decisions were similar to those observed with delay and effort. Thus, the present results provide a solid generalization of the effects that were observed here and there, including in our own studies focusing on risk taking^12,15^. They also help disambiguate some earlier reports using tasks in which different costs were confounded. For instance, in our previous studies, the risk of losing money was associated with the probability of failure in a sensorimotor challenge of varying difficulty, which could arguably mobilize different degrees of effort. The present results show that mood impacts the willingness to accept both risk and effort, even when properly separated in distinct decisions. Note that in the present paradigm, risk was manipulated through the probability of losing something, which corresponds to the common meaning of the word but not to its formal definition in economics (i.e., the variance of expected outcomes). The effects of mood observed here can be contrasted with those of cognitive fatigue that we previously investigated using the same kind of choices^23^. For decisions involving delay and effort, cognitive fatigue induced the effect as bad mood, with participants being less willing to wait and to make effort. However, contrary to mood, cognitive fatigue did not affect choices under risk, presumably because in this case, costly options (watching a lottery) involved no exertion of cognitive control (no wait, no effort).

Among the factors that enabled us to obtain such robust effects is the within-participant comparison, as opposed to between-group comparisons. This is a much more powerful comparison because of the high variability in economic preferences across participants. The drawback is that the manipulation may be detected, which would occasion some demand effects, with participants not really feeling mood changes but understanding this is expected from them, after receiving feedback on their responses. However, in the present study, no participant reported having noticed series of easy and difficult questions, or that a wrong answer could be given positive feedback. Moreover, had they understood the manipulation, it remains unlikely that could have guessed what effect was expected on their economic choices. Note that a direct effect of feedback would rather point to searching compensation, meaning going for larger rewards after negative outcomes (as when gamblers chase after losses), which is the opposite of what was observed. Another factor that helped us demonstrating robust effects is the online trial generation procedure, which neutralized inter-participant variability by tailoring the choices presented to their idiosyncratic indifference points. This procedure avoids taking the time of a full calibration session and adjusts to any change of preference within the test session. It also avoids committing to any specific discount function, using a flexible and agnostic piecewise linear function to approximate indifference curves. Finally, even if it may go against an expected change in choice rate, it provides a direct readout of preference shift with the indifference curve. This procedure may therefore be useful to any study exploring how cost–benefit tradeoff varies across individuals or contexts.

The results are in line with our theory that mood flexibility is adaptive because it helps making decisions to forage when the expected cost–benefit tradeoff is favourable. The idea that mood flexibility may help exploit momentum in reward availability was initially proposed by Eldar and colleagues^18^. We have elaborated on this idea in our own model, dubbed MAGNETO (for Mood As Global Net Expected Trade-Off), which contains a learning function that keeps track of the average costs and benefits associated with foraging actions, and a choice function that determines decisions to forage based on expected costs and benefits^22^. Here, the learning function could not be tested, because we independently manipulated mood with feedbacks that were unrelated to the outcomes of chosen actions. Thus, we only tested the validity of the choice function, in which mood controls a global preference in favour of foraging, i.e. accepting to take risk, spend time and make effort to get more reward, above and beyond the specific features of possible actions. The results fulfilled the prediction that higher mood provides an additive bonus to more rewarded but more costly options, which manifested as both higher choice rate and faster decisions. Note that Eldar’s model would not make this prediction, because it assumes that mood (defined as position on a happiness-to-sadness dimension) only affects learning, not decision-making. Even in recent developments of this model^33^, happiness and sadness are still assumed to affect value learning, while other emotions would modulate action tendencies. Whether they affect learning or decision-making, mood fluctuations may have been adaptive for our hunter-gatherer ancestors, because of spatial and temporal correlations in reward sources and action costs. These correlations stem from natural phenomena like seasonal variations, rewards being scarcer and foraging being more costly during cold and dark winters. In many situations of our modern world, however, mood triggers are unrelated to choice outcomes, as when victory in a sports competition drives economic risk taking. In the latter case, as in our experimental settings, mood impact on decisions may rather be considered as an irrational bias. Thus, the mood bias that might have been selected because it was adaptive in natural environments, must still operate today in a somewhat implicit and automatic manner, since it manifests even in artificial environments where mood triggers and choice outcomes are clearly independent. This does not mean that the bias cannot be counteracted, by reasoning in a more explicit manner about the likely consequences of choice options, or even by simply realizing that current mood may unduly influence decision making.

In our previous studies focusing on how mood fluctuations affect choice under risk, we found, using neuroimaging techniques (fMRI and intracerebral EEG), that positive and negative episodes were associated with opposite activities in specific brain regions (the ventromedial prefrontal cortex, vmPFC, and the anterior insula, aIns). Because the induction procedure was almost identical, similar brain regions may have mediated the effects of mood fluctuations on the different decisions of the present study, with baseline vmPFC activity pushing towards large but costly rewards and baseline aIns activity pushing towards uncostly but small rewards. Here, the effect of mood was additive to that of option attributes, meaning that we cannot say whether good mood amplifies expected rewards or reduces expected costs. This effect may be induced by baseline activity (prior to option display) in mood-related brain regions shifting the neural responses that signal reward and cost levels. Such a mechanism through which pre-choice brain activity influences the neural valuation of choice options has already been reported^24,34–36^. By opposition to a multiplicative effect, an additive effect can be interpreted as mood imposing a default option, which would be selected in the absence of further information about the costs and benefits associated to possible actions. Indeed, in our model, the selection of costly versus uncostly action is entirely determined by mood when the difference in value is null, which may happen either because there is no information or because there is no time to process the information.

Although normal mood fluctuations may be adaptive in some circumstances, they can turn into pathological conditions, as seen in mood disorders^37–39^. Decisions in mood disorders are heavily biased by mood states, with manic patients engaging in ambitious risky projects and depressed patients withdrawing from most actions even those with little cost (according to DSM5 criteria). Thus, the behaviour of patients undergoing depressive and manic episodes may be captured by extreme mood bias parameters in the cost–benefit analysis that drives engagement in behavioural activities^22^. This would accord well with reports that patients with major depressive disorder are less willing to exert effort for reward^21,40–42^, an observation that has been specifically related to the enhancement of subjective effort cost^43^. We note, however, that the time scale of mood disorders (several months, typically) is much different from that of the mood fluctuations induced in the present study (several minutes). Even normal moods occurring in healthy people are usually slower to emerge and vanish (from hours to days), as shown by experience-sampling methods^44,45^. Yet the choice bias in our study was not solely due to the last feedback but rather to mood rating, which integrated a series of feedbacks in the course of positive or negative episode. Thus, the moods induced here may be seen as intermediate cases between emotional reactions (which are restricted to a single trigger) and mood fluctuations (which are prolonged over longer periods). Further studies are needed to assess whether the mood bias also occurs at both shorter and longer time scales. Another limitation is that subjective experience of positive and negative episodes was only reported in a single mood dimension, as the decomposition into valence and arousal components assessed during pilot experiments remained unsuccessful. Future studies will tell whether reported mood is the relevant dimension for decision making or whether it can be refined, for instance into happiness and sadness sub-components. Yet another limitation is that choices were not incentivized in the present main study. However, a randomly drawn subset of selected options were executed in our pilot study where we obtained similar effects of the mood manipulation, suggesting that incentivisation may not be a critical factor. This makes our paradigm usable in clinical settings, where actual delays, efforts and bank transfers are usually difficult to implement, for both practical and ethical reasons.

## Methods

The studies were approved by the Ethics Committee for Biomedical Research of the Pitié-Salpêtrière Hospital, where the experiments were conducted. All experiments followed the guidelines and regulations of the Paris Brain Institute, and were in accordance with the Declaration of Helsinki. In this section, we describe the methodology and materials used for the main study. We refer the reader to the Supplementary Methods S1 for a detailed list of differences with pilot studies 1 and 2.

### Participants and procedure

Participants were recruited through an online database of candidates for behavioural experiments in the greater Paris area. Inclusion criteria were: being 18 years of age or older, being a French native speaker, and not having a history of psychiatric or neurologic disorders.

In total, 104 participants registered to take part in the main study. This study consisted of two sessions done during two visits scheduled approximately two weeks apart. Two participants who did not come back for the second session were excluded from analyses, so our sample comprises 102 subjects. Each session consisted of the current experiment and another independent experiment that will be reported in a separate paper. Participants were paid a fixed endowment of 50€ for participation in both sessions.

The study took place in the PRISME behavioural facility of the Paris Brain Institute. Participants gave informed consent before starting the experiment. They then viewed instructions for the choice tasks and performed 8 example trials. For mental effort, participants were shown a print sample of a text page that would have to be copied. Subsequently, instructions for the quiz task were given and participants played 10 example trials. Importantly, contrary to pilot studies, the choices presented in the main study were not incentivized, and participants were told that, although choices were hypothetical, they should make decisions that best reflect their preferences and that there were no right or wrong answers. This was done to validate a paradigm that would be usable in the clinic, where implementation of choices is usually difficult.

### Task design and apparatus

The quiz task consisted of questions that were drawn from the French version of the ‘Trivial Pursuit’ game. Quiz questions were sorted according to the rate of correct answers given by participants in a previous study^15^ and then divided into categories of relatively easy, intermediate, or relatively hard questions.

In every trial, a quiz question was presented for 3.5 s, followed by 4 possible answers from which one had to be selected within 4.5 s. Hard, easy and intermediate questions were respectively presented during negative episodes, positive episodes and transitions between episodes. The 1 s feedback screen showed a smiling emoji accompanied by a cheerful *ping* sound (positive feedback) or a frowning emoji accompanied by an unpleasant *buzz* sound (negative feedback). A player always received positive feedback when answering correctly, and feedback was always negative when the player did not answer within the time limit. Feedback following an incorrect answer depended on the bias, which could be of 0%, 25%, or 50% according to the experimental condition (negative episode, transition, positive episode, respectively). After receiving feedback, participants made one economic decision between a small uncostly reward and 30€ associated with one of 4 possible costs. The first type of cost featured a delay until payment of up to one year, visualized by a calendar with the number of days to wait coloured in red. The second type featured a risk to lose 10€ if the participant chose to enter a lottery, symbolized by a wheel-of-fortune where the red/green shaded area represented the probability of losing a certain amount / winning 30€. The third type featured a physical effort in the form of up to 12 flights of stairs to climb. The fourth type featured pages to copy from a text in a non-existent language, each page containing 25 lines. The location on screen (left or right) of the costly option was counterbalanced across trials. Each cost type was assigned to one of four consecutive trials, the order being pseudo-randomized such that the same cost could not appear twice in a row. A trial ended with participants rating their mood, by placing a cursor along a continuous scale ranging from “bad mood” to “good mood”. In French, the question was *“Comment je me sens?”* and the labels at the scale extremities were *“de mauvaise humeur”* and *“de bonne humeur”.*

The experiment was conducted on computers with tactile screens, so any selection of quiz answers, choice options, or mood level was done by the touching the appropriate location with the index finger. The task was coded in MATLAB using the PsychToolbox^46^.

### Online trial generation

In order to reveal the impact of mood on decision-making, we needed to present choices for which the preference of the participant was uncertain. To save the time of a calibration session, and to adjust for variations within the test session, we used online trial generation (OTG, see https://github.com/MBB-team/OTG). This procedure updates indifference curves based on observed choices and uses it to select options for upcoming choices.

#### Indifference map

To avoid committing to any a priori discount function that might fail to capture choice data or to generalize across tasks, we used an agnostic model that, in principle, can approximate any shape, under the assumption that the subjective value of reward declines continuously as the cost increases (Fig. 3a). The cost range was divided into 5 bins, equally spaced between 0% (no cost) and 100% (maximum cost). The subjective value function was described using a piecewise linear model, where (i) discounted value is assumed to decrease linearly within cost bins, and (ii) discounted value at edges of a given cost bin is constrained to match that of adjacent bins. It is therefore defined by 5 slope parameters ${k}_{1},{k}_{2},\dots ,{k}_{5}$. For example, in bin 1 where cost $C\in ]0, 0.2]$, the expressions for the local linear functions are as follows:

7 $${V}_{uncostly}=r+{b}_{0}$$

8 $${V}_{costly}=1-{k}_{1}\cdot C$$

In our design, options for which *C* > 0 are associated with a fixed maximum reward (equal to 30€ in practice), which is by definition the 100% reward (*R* = 1). Options for which *C* = 0 are the small rewards noted *r* (with *r* < *1*). Indifference points (for which costly and uncostly options have the same value) are therefore defined by an equivalence between small reward r and cost level C, which is for bin 1:

9 $$r=1-{b}_{0}-{k}_{1}\cdot C$$

Here, for the first bin, the intercept is *1* − *b*_*0*_, but in other bins, the intercept of the local linear function must be calculated at the bin edges (see the Supplementary Information S1 for full mathematical details). Note that *b*_*0*_, the intercept of the first bin, corresponds to the bonus added to uncostly options, so it is equivalent to the bias of the softmax function used in the choice model described below. The free parameters were constrained to be positive and fitted with prior means of 1 for slopes *k*_*i*_ and on 0 for *b*_*0*_.

For each point of the space defined by the small reward and cost level, we modelled the probability *P* of a costly choice with a softmax function (as expressed in Eq. 5)*.* This enables us to derive an indifference score, which we define as the distance between the modelled choice probability and 50%, normalized to span the [0,1] range:

10 $$Indifference=\frac{0.5-|P-0.5|}{0.5}.$$

In turn, the expected indifference of any combination of reward and cost can be scored every time a new choice is observed (Fig. 3b). This provides a map of expected indifference on a 50 × 50 grid that combines cost levels (from 2 to 100%) and small rewards (from 0.33 to 99.67%, i.e. from 0.10€ to 29.90€).

#### Option selection

The indifference map was used to select the options presented in the next choice trial. First, a probability density distribution was generated by vertically summing indifference for each cost level, and normalizing across the 50 obtained sums. The next cost level was selected by randomly sampling from this distribution, thus, cost levels featuring more combinations close to indifference were more likely to be selected. Then, the small reward for the uncostly option was selected such that it equals the selected cost combined with the big reward (30€), given the current estimate of the indifference curve. Finally, the two selected options were presented for the participant to make a choice.

A Bayesian parameter inference scheme was used to update the parameter estimates of the indifference curves based on the observed choices (see the Supplementary Information S1 for relevant mathematical details). As the experiment proceeds, parameter estimates get more precise because more trials are included. However, the OTG algorithm must allow for changes in preference over time, which could be caused by moods. For this reason, we only included the 20 most recent choices as observations when fitting the model. Conversely, to allow for a minimum of observations, the online parameter estimation started only after the first three choices had been made.

#### OTG performance

Before adopting OTG, we compared its performance to that of alternative sampling methods using numerical simulations (Fig. S3a). The first alternative to OTG was a predefined square grid sampling over the small reward x cost level space, using a lattice of 9, 16, 25, 36, or 64 trials, the latter corresponding to the total amount of choices per type in the main experiment. Since including more trials enhances model fit, independent of their location in the reward/cost space, we introduced a second alternative, which consists in randomly sampling more or less reward/cost combinations. This serves as a benchmark experimental design for later performance comparisons. We decided to run a number of simulations that was three orders of magnitude greater than the number of free parameters^13^, so the analysis comprised 13^3^ = 2197 simulations. To reproduce the properties of our experimental data, each simulation featured a different set of parameters that were randomly drawn from the empirical distribution of parameter values in the experimental population. At each trial, the three pairs of differentially sampled options were presented to a dummy agent that behaved according to the winning choice model [see Eqs. (11)–(18) below]. The trial-by-trial simulated choice sequence then entered a model inversion procedure implemented in the VBA toolbox^26^. Post-hoc choice predictions were then used to derive the so-called balanced accuracy (by averaging the predictive accuracy obtained for costly and uncostly choices). This goodness-of-fit measure was the first performance metric used for comparison between sampling procedures (Fig. S3b, left). OTG outperformed random sampling for all numbers of included trials, and did better than grid sampling whenever the lattice contained 16 trials or more. The second metric for comparison between sampling methods was the posterior variance of parameter estimates (Fig. S3b, centre). The reasoning here is that procedures yielding lower posterior variance effectively provide more reliable parameter estimates. Again, OTG outperformed random sampling at any number of included trials, and did better than grid sampling after 25 or more trials were included. A third performance metric was the recoverability of parameters of interest (Fig. S3b, right). In particular, our main parameter of interest was the bias that mood exerted on choices. We thus compared the correlation between simulated and recovered *β*_*Mood*_ between sampling methods. This was the 14th free parameter, so we ran 14^3^ = 2744 simulations. Again, the OTG procedure outperformed the other sampling methods, showing better recoverability, no matter how many trials were included.

### Data collection

Model-free dependent variables were observed choice (costly or uncostly) and response time (RT). Trials with extreme RT were excluded (8.9% per participant in total), keeping only trials with 0.75 s < RT < 10 s and within the median ± 3 SD interval. The session number (categorical variable) and trial number within session (continuous variable) were regressed out. To better gauge the momentary willingness to accept costly options, we calculated at each trial the area under the curve (AUC) of indifference estimated by the OTG procedure. AUC was computed by applying the trapezoidal rule to each of the 50 cost levels for which the equivalent small reward was larger than zero. AUC was then z-scored per session and interpolated separately for each cost type (because only one cost type was presented at every trial). Finally, episode trial effects and first-episode order effects were regressed out.

Choice rate, corrected RT, and AUC were then compared between positive and negative episodes using paired two-tailed t-tests at the group level. Regression models meant to explain dependent measures (choice or AUC) with manipulated factors (feedback or mood) were estimated at the individual level and regression estimates were then compared to zero at the group level using one-sample two-tailed t-tests.

### Choice model selection and comparison

In the definition of a model space for the four types of costs, we started with discount functions that generate subjective values. For risk discounting, we chose a standard expected value model, with an additive cost term scaled to the probability of losing. For delay discounting, we opted for an exponential decay function, because it was shown to best fit inter-temporal choices in our previous work^23,47^ when combined with an additive bonus for immediate rewards. For effort discounting (both physical and mental), we selected a parabolic decay model that has become consensual in recent literature^48–50^.

These functions had only one parameter, their discount factor *k*, a weight parameter that was specific to the cost type (because risk, delay and effort levels were expressed in different units). The first extension of these discount functions was the inclusion of a weight on reward *k*_*Rew*_ that was shared across all cost types (because reward was always a monetary amount in euros). The second extension was the inclusion of power parameters *γ* on the different costs, in order to capture more extreme curvatures of the discount function. Note that standard discounting models simply reduce to fixing the power to 1 for delay and risk discounting, and to 2 for mental and effort discounting.

To generate choice probabilities from decision values, we used a softmax function with an inverse choice temperature *β*_*1*_ (controlling choice consistency), and an additive bias term *β*_*0*_ (controlling the attraction of uncostly rewards). Both parameters could either be shared across cost types (one unique parameter), or specific to each cost type (4 different parameters), or absent altogether.

When including all parameters, the extended discount and choice functions were the following:

For delay:

11 $${V}_{D}={k}_{Rew}\cdot Rew\cdot \mathrm{exp}\left(-{k}_{D}\cdot {D}^{{\gamma }_{D}}\right)$$

12 $$P\left(delayed\right)= {\left(1+\mathrm{exp}(-{\beta }_{1,D}\left({V}_{D,costly}-{V}_{D,uncostly}\right)-{\beta }_{0,D})\right)}^{-1}$$

For risk (of losing *L*):

13 $${V}_{R}={k}_{Rew}\cdot Rew\cdot P-{k}_{R}\cdot L\cdot {(1-P)}^{{\gamma }_{R}}$$

14 $$P\left(risky\right)= {\left(1+\mathrm{exp}(-{\beta }_{1,R}\left({V}_{R,costly}-{V}_{R,uncostly}\right)-{\beta }_{0,R})\right)}^{-1}$$

For physical effort:

15 $${V}_{PE}={k}_{Rew}\cdot Rew-{k}_{PE}\cdot {PE}^{{\gamma }_{PE}}$$

16 $$P\left(effortful\right)= {\left(1+\mathrm{exp}(-{\beta }_{1,PE}\left({V}_{PE,costly}-{V}_{PE,uncostly}\right)-{\beta }_{0,PE})\right)}^{-1}$$

For mental effort:

17 $${V}_{ME}={k}_{Rew}\cdot Rew-{k}_{ME}\cdot {ME}^{{\gamma }_{ME}}$$

18 $$P\left(effortful\right)= {\left(1+\mathrm{exp}(-{\beta }_{1,ME}\left({V}_{ME,costly}-{V}_{ME,uncostly}\right)-{\beta }_{0,ME})\right)}^{-1}$$

The full model space contained 36 different combinations that are listed in Table S4.

Models were inverted using the Variational Bayesian Analysis (VBA) toolbox (available at https://mbb-team.github.io/VBA-toolbox). It relies on a variational approach to Bayesian inference, thereby being orders of magnitude more efficient than sampling-based approaches^26^. Inputs to VBA were prior parameter distributions, trial-by-trial option features and choices. The weights *k* on reward and costs, the power parameters γ and inverse choice temperatures *β*_*1*_ were constrained to be positive and their prior distributions were centred on 1. Choice bias *β*_*0*_ could be any real number and its prior distribution was centred on 0. Outputs from VBA were the posterior distribution over parameter estimates and model evidence, which provides a tradeoff between accuracy (goodness of fit) and complexity (number of free parameters).

For Bayesian model selection^51,52^, group-level random-effects analyses were applied to individual log-model evidence, with the underlying assumption that different models could be used by different participants. These analyses provided the expected frequency with which each model prevails in the population, as well as the exceedance probability that quantifies the probability that a given model is more frequent than all the others within the considered model set. The winning model (see description in main text), from the comparison between the 36 possibilities, reached an exceedance probability of 94% (see Table S4).

### Supplementary Information

Supplementary Information.

## Data Availability

All data needed to evaluate the conclusions in the paper are present in the paper and/or the Supplementary Materials S1. The raw data (MATLAB datasets) can be provided by Roeland Heerema pending scientific review and a completed material transfer agreement. Requests for the data should be submitted to: mathias.pessiglione@gmail.com. The toolbox used for online trial generation can be accessed at https://github.com/MBB-team/OTG.
