# Supplementary material for: Mood fluctuations shift cost–benefit tradeoffs in economic decisions
Source: Sci Rep. 2023 Oct 24;13:18173. doi: 10.1038/s41598-023-45217-w (PMC10598198; doi:10.1038/s41598-023-45217-w)
Supplement: Supplementary file 1 — Supplementary Information. [file 41598_2023_45217_MOESM1_ESM.docx]

Supplementary Materials for

**Mood fluctuations shift cost/benefit trade-offs in economic decisions**

Roeland Heerema* *et al.*

*Corresponding author. Email: [roelandheerema@hotmail.com](mailto:roelandheerema@hotmail.com)

**This PDF file includes:**

Supplementary Information

Figs. S1 to S3

Tables S1 to S4

**Supplementary Information**

**Experimental procedures for pilot studies**

Two pilot studies with smaller sample sizes (see **Table S1**) preceded the main experiment that is described in the main text. All relevant differences in experimental procedures are described here.

Participants performed the experiment on the same equipment as in the main study, but they used keyboard buttons to play the quiz or make economic choices, and the mouse for ratings. Before the experiment, instructions and examples were shown for the quiz game and the economic choice tasks. Critically, choice options during the experiment were not selected using OTG in study 1. The experiment was preceded by a choice calibration procedure for each cost type (see below) in both pilot studies. It was omitted in the main study because it was deemed redundant in study 2.

Contrary to the main experiment, economic choices in the experimental sessions of studies 1 and 2 were incentivized. This means that final payoffs were dependent on participants’ choices (see **Table S1**). In study 1, the total payoff entirely depended on three trials randomly drawn from all the choices a participant had made. The selection of implemented trials was actually pseudo-randomized such that all participants would receive a payoff between 30€ and 45€. One trial featured a lottery, where an animated wheel-of-fortune was spun if the risky option had been chosen. One trial featured a delay so that if the delayed option was selected, that part of the payoff would be wired by bank transfer after the indicated delay (the rest was paid in cash at the end of the experiment). The last drawn trial featured a physical effort that differed with respect to the subsequent studies: in case the effortful option was drawn, a participant had to spend 15 minutes on the fitness bike, pedalling at the selected effort level. Participants experienced the different effort levels in a training session on the bike conducted prior to the experiment. The different effort levels were adjusted to individuals depending on the maximum power they could sustain. In study 2, the total payoff consisted of a fixed reward of 20€, plus a variable reward that depended on one trial that could be drawn from any of the four cost types also used in the main study. When the implemented trial featured a physical effort, participants climbed the required amounts of stairs in the stairwell of the Paris Brain Institute. Although the costly option of a mental effort choice was not drawn for any of the 21 subjects, participants were told that they would take home a paper copy of the non-existent language text, printed in a font that could not be read by text recognition software when scanned. They would receive their payoff once they correctly copied the required number of lines and e-mailed it to us. The selection and animation of risky choices was biased such that participants’ variable reward was never negative. In doing so, their final payoff would always be between 20€ and 50€.

Mood induction

The design of the mood-inducing episodes varied across studies (for an overview, see **Table S2**; for a visualization, see **Figure S1a**). Relevant differences with respect to the main study are depicted below.

There were two key differences in study 1. First, there was a third ‘neutral’ condition, in which quiz questions were presented with multiple answers as per usual, but the correct answer was already highlighted. The participant simply had to click on the correct answer and received no feedback. Second, every trial featured a rating of well-being that could be any of four dimensions: happy (‘content’ in French), sad (‘triste’), calm (‘calme’), and stressed (‘tendu’). Participants rated to which extent they felt as such, from ‘not at all’ to ‘totally’. Each of these dimensions was presented once per set of four subsequent trials, and no two subsequent trials featured ratings of the same dimension. Note that this implied that interpolation was required to obtain a time-resolved rating for each of these scales (see **Figure S1b**). In the analyses where relations between mood and choice are estimated, “mood” is constructed post-hoc by taking the difference between the interpolated and standardized ratings of happiness and sadness. In study 2, the rated mood dimension was the same as in the main study, but participants were only prompted to rate their mood in 2/3 of trials. These ratings either followed after the quiz feedback as in the main experiment (1/3 of trials) or only after the economic choice (1/3 of trials).

Both studies 1 and 2 were done in one sitting, although in study 1 there was a short break after every 3 episodes. Study 1 featured 9 episodes, organized in 3 sets of 3 where each condition was featured once and no two subsequent episodes were of the same condition. Study 2 featured 6 episodes where positive and negative episodes alternated with each other and the first condition was randomly determined. The staircase-wise organization of feedback bias also differed among studies. In study 1, 21 trials were of the maximum/minimum bias, and an episode was only preceded by 7 transition trials. In study 2, the bias during an episode was gradually increased/decreased by increments/decrements of 12.5% every 6 trials. Episodes were preceded and followed by 3 transition trials.

Choice task

The overview of choice task details specific to the different studies are listed in **Table S3.**

The conception of choice trials was largely the same in the main study and pilot study 2. There were differences, however, with pilot study 1. After study 1, the maximum reward was increased from 15€ to 30€, the potential loss in risky trials was decreased from 15€ to 10€. The maximum delay was reduced from 2 years to 1 year and mental effort was added as a cost type (making 4 the number of choices per trial instead of 3). As mentioned above, the nature of the physical effort was changed from pedalling on a fitness bike to climbing flights of stairs.

A notable difference in the main study compared to pilot studies 1 and 2 was the absence of a choice calibration session. The purpose of a choice calibration is to find the individual’s “baseline” indifference points. In study 2, the OTG algorithm was used to calibrate choice preferences in 60 trials per cost type. The obtained model parameters were subsequently used as priors for the OTG during the experiment. As calibration took a long time (about 20 min, excluding explanations, instructions on screen, and example trials), it was omitted in the main study, which used uninformed priors for OTG (as illustrated by the straight line in **Figure 3a**). In study 1, there was also a choice calibration of 40 trials per cost type that employed a rudimentary form of OTG. Here, we pre-committed to a set of discount functions that, incidentally, corresponded to the model formed by **Equations 1-3**, except that the choice function only featured one shared bias and inverse temperature. During calibration, costs were drawn from an evenly spaced interval in a random order, and rewards were determined by a criterion of maximum efficiency, which means, for the given cost, selection of the corresponding small reward (for the uncostly option) that would yield the largest decrease in posterior variance. A full trial list for the experiment following the calibration was then generated. Cost levels were sampled from a uniform distribution over the positive range of the calibrated indifference curve. The corresponding small rewards for uncostly options were sampled according to the following rule: of the 28 trials in an episode (including the 7 prior transition trials), 10 were sampled at indifference, 12 were close (at 10% above/below), 6 were far (20% above/below). After fitting the winning model of the main experiment (**Equations 1-3; 6**), the distribution of decision values in study 1 could be compared to that of the main experiment, and indeed the variance around indifference was much larger. Although the differences between designs are numerous and sample sizes are of a different order, we argue that having a distribution of trials more closely centred around DV = 0 has contributed to a heightened sensitivity to mood effects on choice.

**Comparison of protocols used in pilot studies and in the main study**

***Table S1. Demographic and payoff details per study.***

|  | Study 1 | Study 2 | Main study |
| --- | --- | --- | --- |
| *N* (female/male) | 25 (16/9) | 21 (13/8) | 102 (76/26) |
| Age: mean (*SD*) | 26.36 (3.74) | 25.19 (6.02) | 32.51 (16.34) |
| Payoff: mean (*SD*) | 35.25€ (3.74€) | 35.40€ (11.52€) | 50€ (0) |

*Note that choices were incentivized in pilot studies but not in the main study.*

***Table S2. Design specifics of mood induction in the different studies.***

|  | Study 1 | Study 2 | Main study |
| --- | --- | --- | --- |
| Episode conditions | pos./neg./neutral | pos./neg. | pos./neg. |
| Number of episodes | 9 (3 per cond.) | 6 (3 per cond.) | 8 (4 per visit, 2 per cond.) |
| Episode condition order | interleaved | interleaved | random |
| Trial totals | 252 trials  (63 per cond.) | 144 trials  (54 per cond.) | 256 trials  (128 per visit, 72 per cond.) |
| Rating totals | 252 ratings (63 per dim.) | 96 ratings | 256 ratings (128 per visit) |

***Table S3. Design specifics of economic choices in the different studies.***

|  | Study 1 | Study 2 | Main study |
| --- | --- | --- | --- |
| Different choice types | 3 (D/R/PE) | 4 (D/R/PE/ME) | 4 (D/R/PE/ME) |
| Max. reward (costly option) | 15 € | 30 € | 30 € |
| Fixed loss (risk choices) | 15 € | 10 € | 10 € |
| Max. delay | 2 years | 1 year | 1 year |
| Physical effort | Fitness bike | Staircases to climb | Staircases to climb |
| Mental effort | N/A | Pages to copy | Pages to copy |
| Calibration | Yes (40 trials/type) | Yes (60 trials/type) | No |
| Online trial generation | No | Yes (calib. + exp.) | Yes (exp. only) |
| N choices | 756 (252 per type) | 576 (144 per type) | 256 (128 per visit, 64 per type) |
| Implementation | Yes (1 trial/type) | Yes (1 trial overall) | No |

*Abbreviations used: D (delay), R (risk), PE (physical effort), ME (mental effort)*


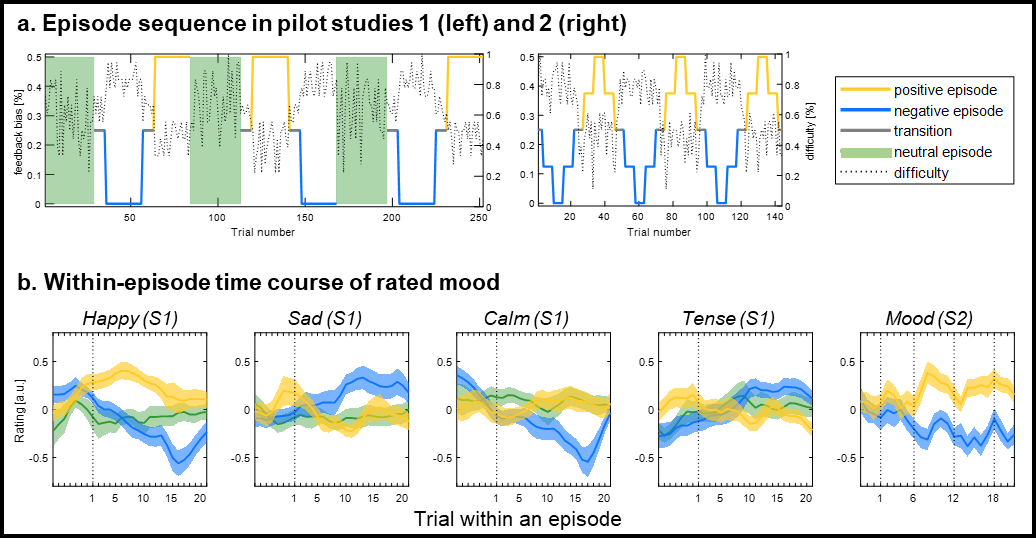


***Figure S1. Mood fluctuations in pilot studies 1 and 2.***

***(a)*** *Experimental session in one example participant. Coloured solid lines indicate feedback bias (proportion of incorrect answers given a positive feedback) and dotted lines denote quiz question difficulty (rate of incorrect answers obtained from an independent group). In pilot study 1 (left), there were 9 episodes of 3 kinds: positive, negative, or neutral. In the neutral condition, no feedback was given and question difficulty could be of any level. Pilot 2 featured only positive and negative episodes but there were 5 feedback bias levels.****(b)*** *Effect of feedback on mood. Solid lines show the rated mood averaged across participants, shaded areas represent inter-participant standard errors around the mean. Ratings were standardized and epoched around the positive and negative episodes. In pilot 1 (4 left subfigures), participants rated to which extent they felt happy, sad, calm, or tense. The epochs shown include positive and negative episodes (50% and 0% feedback bias), plus the 7 pre-episode transition trials (25% bias). In pilot 2 (rightmost subfigure), participant rated their mood. The positive and negative conditions include all trials in which feedback bias was respectively above 25% (either 37.5% or 50%) and below 25% (either 12.5% or 0%). The epochs shown include the 3 transition trials (25% bias) before and after episodes.*


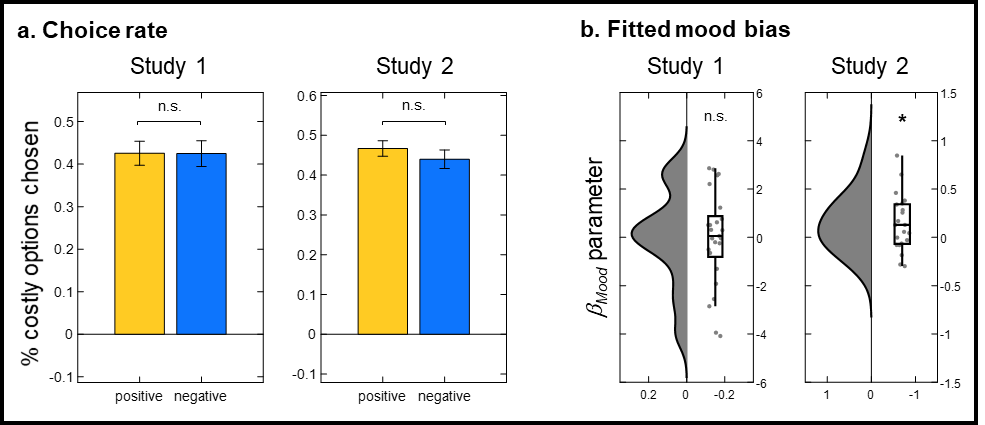


***Figure S2. Effects of mood induction on choice behaviour in studies 1 and 2.***

***(a)*** *Comparison of costly choice rates between positive and negative episodes. Error bars are inter-participant standard errors of the mean.*

***(b)*** *Individual mood bias parameters and overall distribution. A positive parameter indicates an increased tendency to choose the costly option along with improved mood, and vice versa for the uncostly option as mood deteriorates. The winning model from the main study was applied to the choice datasets of studies 1 and 2. Note that, strictly, mood was not rated in study 1 but constructed post-hoc by taking the difference between rated happiness and sadness. The graphs are generated with the Raincloud Plot toolbox (50).
* p < 0.05; n.s. not significant.*

**Supplementary Information about the main study**

Main study: Model comparison and recovery analysis

The final model from the main study includes a weight on reward, 4 discount factors, 4 inverse choice temperatures, 4 choice biases, and a weight on mood to modulate the choice bias. The latter parameter was added to the winning model from a model comparison across a space of 36 models (**Table S4**). An alternative to having mood modulate the choice bias is to have a multiplicative effect of mood in the discount function, where it would affect the weight on reward for the generation of subjective value. In a direct competition between these two alternatives, the Bayesian model comparison clearly favoured the additive mood bias model (estimated model frequency of 66.9 vs. 36.1; exceedance probability of 99.9% vs. 0.1%).

For the final model, we tested whether the included parameters were well identifiable given the observed data. To this end, we ran a simulation-recovery analysis by sampling parameters from the observed empirical distribution. This model has 14 free parameters, so we ran 14^3^ = 2744 simulations. We generated 256 trials (64 per choice type) with OTG and simulated decisions given the sampled parameters and given simulated moods, sampled from a Gaussian function fitted to the empirical distribution of rated moods. Next, we inverted the model on the simulated choice data and retained the fitted parameters. These parameter estimates were standardized and regressed on the standardized simulated parameters in a multivariate analysis. By doing so, the variance of the estimated parameters that is explained by the simulated parameters could be approximately assessed as the square of the regression weights. Each fitted parameter was best identified by its simulated counterpart, with a R^2^ that was greater than the sum of all other parameters’ R^2^. In addition, alternative ways of generating choices (grid sampling and random sampling, see **Methods**) were compared to OTG in a simulation-recovery analysis (**Figure S3**). Specifically, the identifiability of our key mood bias parameter *β_Mood_* was better recovered by OTG than with other sampling methods, whatever the number of trials that were included in the analyses.

***Table S4. Full model space and comparison results.****The weight parameter on reward was unique and could be applied or not to all discount functions that generate subjective values. The power parameters were specific to each type of cost. When not included, the power was fixed to 1 for delay and risk, and to 2 for physical and mental effort (which corresponds to parabolic discounting). The inverse temperature and choice bias parameters could be either omitted, shared across cost types, or specific to each cost type. Estimated model frequency is the expected proportion of participants for which the considered model is more plausible than the others. Exceedance probability is the likelihood that the considered model offers a better explanation of choice data than all the others.*

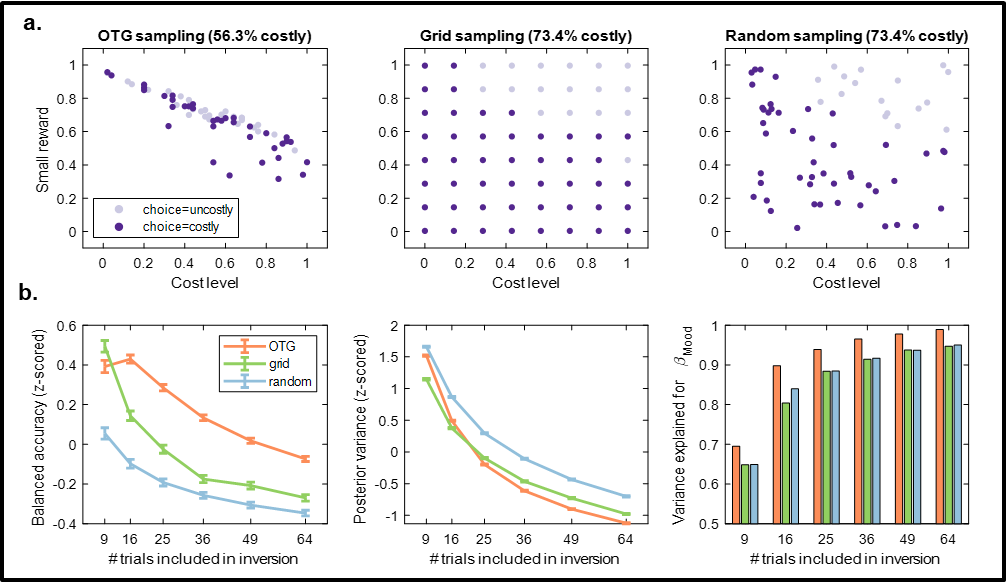


***Figure S3. Comparison of sampling approaches to the generation of choice options.***

***(a)*** *Illustration of the three sampling methods.The two colors indicate the decisions by an arbitrary simulated agent that is faced with choices generated under the three sampling rules. The agent’s costly option choice rate is indicated within brackets.*

***(b)*** *Comparison of performance measures between sampling methods. Graphs show performance as a function of the number of trials included in the simulation/recovery analysis. Balanced accuracy is the proportion of correctly predicted choices calculated separately for the two possible decisions and then averaged (note, in order to be able to compare balanced accuracy across sampling methods, the generated trials from all three methods were pooled). Posterior variance is an average over fitted parameter distributions. Variance explained is the recoverability of the key mood bias parameter. Error bars are standard errors of the mean calculated across simulations.*

Main study: details of the OTG algorithm

The online trial generation algorithm updates, on a trial-by-trial basis, an estimation of the utility (cost-discounting) function that underlies participants’ choices. In what follows, we derive the main steps of its algorithmic derivation, which relies upon a piecewise linear approximation of participants’ discount function (see **Methods** section).

***The Laplace approximation to Bayesian inference***

Consider observed binary choices $y\in\left\{ 0,1 \right\}$ that are generated from the comparison of the (parameterized) utility $u(\theta)$ of their corresponding outcomes. In our context, this reduces to comparing the values of the costly and uncostly options (see Equations 7-8). We assume that, given utility parameters $\theta$, the probability of choosing option 1 monotonically increases with the value difference $\Delta u\left( \theta\right)$:

1. $P\left( y=1 | \theta\right)=S(\Delta u\left( \theta\right))$

where $S\left( \cdot\right)$ is the standard sigmoid mapping from Equation 5. A trial $t$, the conditional probability to observe *either* option being chosen is thus given by:

1. $P\left( y_{t} | \theta\right)=P\left( y_{t}=1 | \theta\right)^{y_{t}}\cdot\left( 1-P\left( y_{t}=1 | \theta\right) \right)^{1-y_{t}}$

Given utility parameters $\theta$, choices are independent from each other. This implies that the probability of observing any particular sequence of observed choices $y=\left\{ y_{1},\ldots,y_{T} \right\}$ can be written as:

1. $P\left( y | \theta\right)=\prod_{t=1}^{T} P(y_{t}|\theta)$

Equation 21 specifies the likelihood of utility parameters $\theta$.

Within a Bayesian framework, the likelihood enters Bayes’ rule to yield the posterior probability density function $P\left( \theta\right|y)$ over utility parameters:

1. $P\left( \theta\right|y)=\frac{P\left( y | \theta\right)P(\theta)}{P(y)}$

where $P(\theta)$ is the so-called prior probability density function, which captures the information one has over utility parameters before having observed the choice data. Without loss of generality, we assume that they are normally distributed with mean vector $\mu_{0}$ and covariance matrix ${}_{0}$ :

1. $P\left( \theta\right) = N(\mu_{0}, {}_{0})$

where $N\left( \cdot,\cdot\right)$ denotes the Gaussian probability density function.

Importantly, this does not imply that the posterior $P\left( \theta\right|y)$ is Gaussian as well. In fact, the exact form of the posterior probability density function actually depends upon the way the utility function is parameterized. Nevertheless, one can provide a closed-form approximation to $P\left( \theta\right|y)$, which simplifies the statistical inference on utility parameters $\theta$. In what follows, we rely on the so-called Laplace approximation (*1*), which summarizes the posterior distribution in terms of a Gaussian probability density function with mean $\mu$ and variance :

1. $P\left( \theta| y \right) \approx N\left( \mu, \right)$

As we will see, this enables us to derive analytical belief update rules for the posterior moments.

Recall that if $\theta$ is a Gaussian random variate, then its log probability density function is simply a quadratic function of $\theta$. The Laplace approximation thus consists in finding the quadratic function of $\theta$ that best matches the log posterior probability density function.

Let us first approximate the log posterior probability density function $f\left( \theta\right)=\log P\left( \theta| y \right)$ using a second-order Taylor expansion:

1. $f\left( \theta\right)\approx\underset{\tilde{f}\left( \theta\right)}{\underbrace{f\left( \mu\right)+\left. \frac{\partial f}{\partial\theta} \right|_{\mu}\left( \theta-\mu\right)+\frac{1}{2}\left( \theta-\mu\right)^{T}\left. \frac{\partial^{2}f}{\partial\theta^{2}} \right|_{\mu}(\theta-\mu)}}$

where the approximation is performed around some arbitrary parameter setting $\mu$. We call $\tilde{f}\left( \theta\right)$ the truncated Taylor expansion of $f\left( \theta\right)$. By construction, it is a quadratic function of $\theta$, whose first- and second-order derivatives are given by:

1. $\frac{\partial\tilde{f}}{\partial\theta}= \left. \frac{\partial f}{\partial\theta} \right|_{\mu}{+\left. \frac{\partial^{2}f}{\partial\theta^{2}} \right|}_{\mu}(\theta-\mu)$
2. $\frac{\partial^{2}\tilde{f}}{\partial\theta^{2}}= \left. \frac{\partial^{2}f}{\partial\theta^{2}} \right|_{\mu}$

The shape of $\tilde{f}\left( \theta\right)$ is entirely determined by the expansion point $\mu$, at which the derivatives of $f\left( \theta\right)$and of its truncated Taylor expansion exactly match. Thus, the Laplace approximation is tightest when $\mu$ is set such that the mode $\mu^{*}$ of $\tilde{f}\left( \theta\right)$ coincides with the mode of $f\left( \theta\right)$.

Note that, by definition, $\mu^{*}$ is such that:

1. $\left. \frac{\partial\tilde{f}}{\partial\theta} \right|_{\mu^{*}}=0= \left. \frac{\partial f}{\partial\theta} \right|_{\mu}{+\left. \frac{\partial^{2}f}{\partial\theta^{2}} \right|}_{\mu}(\mu^{*}-\mu)$

where we have made use of Equation 26 to derive the right-hand term of Equation 28.

Equation 28 now implies that $\mu^{*}$ is given by:

1. $\mu^{*}=\mu\underset{\Delta\mu}{\underbrace{-\left( \left. \frac{\partial^{2}f}{\partial\theta^{2}} \right|_{\mu} \right)^{-1}\left. \frac{\partial f}{\partial\theta} \right|_{\mu}}}$

where $\Delta\mu=\mu^{*}-\mu$ is but the difference between the current expansion point $\mu$ and the mode of $\tilde{f}\left( \theta\right)$. Note that $\Delta\mu$ is proportional to the gradient ${\partial f}/{\partial\theta}$ of the exact log posterior. Thus, one can progressively tighten the Laplace approximation by iteratively re-performing the Taylor expansion around a “corrected” expansion point, which is positioned at the previous mode estimate:

1. $\mu\leftarrow\mu+\Delta\mu$

The ensuing so-called Gauss-Newton algorithm modifies the expansion point until it approaches the mode of the exact log posterior $f\left( \theta\right)$, where the gradient ${\partial f}/{\partial\theta}$ tends towards zero. Practically speaking, the expansion point $\mu$ is initialized at the prior mean $\mu_{0}$, and Gauss-Newton corrections are iterated until an arbitrary convergence criterion is reached (here whenever the relative change in parameter estimate $\left\| {\Delta\mu}/\mu\right\|$ falls below 1%). At this point, the expansion point matches the mode of the posterior probability density function, which provides an estimate of utility parameters.

***The Gauss-Newton algorithm for a piecewise linear discount function***

From Equations 19-21, the exact log-posterior $f(\theta)$ is given by:

1. $f\left( \theta\right)=\log P\left( \theta\right)+ \sum_{t=1}^{T} \left[ y_{t}\log S\left( \Delta u \right)+\left( 1-y_{t} \right)\log(1-S\left( \Delta u) \right) \right]$

Equation 31 now provides the first- and second-order derivatives of $f(\theta)$ (Daunizeau 2017):

1. $\frac{\partial f}{\partial\theta}= -{}_{0}^{-1}\left( \theta-\mu_{0} \right)+ \frac{\partial\Delta u}{\partial\theta}\sum_{t=1}^{T} \left[ y_{t}-S\left( \Delta u \right) \right]$
2. $\frac{\partial^{2}f}{\partial\theta^{2}}= -{}_{0}^{-1}-\sum_{t=1}^{T} \left[ S\left( \Delta u \right)\left( 1-S\left( \Delta u \right) \right) \right]\frac{\partial\Delta u^{T}}{\partial\theta}\frac{\partial\Delta u}{\partial\theta}$

Evaluating these expressions at the current expansion point and inserting them into Equation 29 yields the Gauss-Newton correction term $\Delta\mu$ . The above derivations apply to any binary choice model where a decision $y_{t}$ at trial $t$ depends upon a parameterized utility function $u\left( \theta\right)$. However, evaluating Equations 32 and 33 at the current expansion point requires the specification of the trial-by-trial value difference $\Delta u\left( \theta\right)$ and of its gradient ${\partial\Delta u}/{\partial\theta}$.

In our context, the utility function $u\left( \theta\right)$ captures how costs (i.e. risk, delay or effort) discount prospective rewards using a piecewise linear mapping (see **Methods**). It has 6 native parameters: five slopes $k_{1-5}$ and uncostly-option bias $b_{0}$, which can here be interpreted as intercept $(1-b_{0})$ of the discount function (see **Figure 2*Figure*** ). More precisely, the cost space is divided into *n* bins, the edges of which are $[x_{0},x_{1,\ldots},x_{n}]$. In other terms, the costs of bin *m* lie within the range $\left] x_{m-1},x_{m} \right]$. In this study, there were *n = 5* bins and upper edges had values *x_m_ = 0.2 × m*. The local discounting function in each bin is linear, but with its own specific slope (and its bounds are constrained to match those of the discounting function in the adjacent bins), hence the piecewise linear mapping. For example, if $C_{t}\in\left] x_{0},x_{1} \right]$, then the value of the costly and uncostly options can be rewritten as (see Equations 7 and 8 in the Methods section):

1. $V_{uncostly}=r_{t}+\underset{b_{0}}{\underbrace{\exp\left( \tilde{b}_{0} \right)}}$
2. $V_{costly}=1-\underset{k_{1}}{\underbrace{\exp\left( \tilde{k}_{1} \right)}}\cdot C_{t}$

where we have constrained the native parameters to be positive through exponential mappings of dummy parameters $\left\{ \tilde{b}_{0},\tilde{k}_{1} \right\}$. Note that costs and rewards (which are sampled by the online trial generation algorithm) are normalized, such that the costly-option reward equals 1 and the uncostly-option reward $r_{t}<1$.

If $C_{t}\in$ $\left] x_{m-1},x_{m} \right]$, then the value difference $\Delta u\left( \theta\right)$ can simply be obtained by subtracting the value of the uncostly option from the value of the costly option, as follows:

1. $\Delta u\left( \theta\right)=r_{t}-1+\exp\left( \tilde{b}_{0} \right)+\sum_{i=1}^{m-1} \exp\left( \tilde{k}_{i} \right) \left( x_{i}-x_{i-1} \right)+exp(\tilde{k}_{m})(C_{t}-x_{m-1})$

Equation 36 now provides the remaining gradients ${\partial\Delta u}/{\partial\theta}$ w.r.t. $\theta=\left\{ \tilde{b}_{0},\tilde{k}_{1},\ldots,\tilde{k}_{6} \right\}$:

1. $\frac{\partial\Delta u}{\partial\tilde{b}_{0}}=b_{0}$
2. $\frac{\partial\Delta u}{\partial\tilde{k}_{i}}=\left\{ \begin{aligned} k_{i}\left( x_{i}-x_{i-1} \right) if i<m \\ k_{i}\left( C_{t}-x_{m-1} \right) if i=m \\ 0 if i>m \end{aligned} \right.$

This closes the derivation of our Gauss-Newton algorithm for piecewise linear utility functions.

Numerical simulations show that the ensuing Gauss-Newton eventually provides a very efficient mean of localizing the mode of the posterior probability density function $P\left( \theta| y \right)$, which, in most cases, is immune to local minima issues. However, Gauss-Newton corrections sometimes oscillate over iterations, without reaching convergence. This is why we arbitrarily set the maximum number of iterations to be 200, at which point parameter estimates are not updated and the previous posterior modes are retained. This kind of issues arise because of some unfortunate sampling of the cost level and small reward. In such cases, we thus apply a heuristic to update the sampled small reward. The idea is that if the uncostly option is chosen many times within a bin, then the sampled rewards for the uncostly option are too high and must be adjusted downward. Conversely, if the costly option is chosen many times within the bin, then the sampled rewards for the uncostly option are too low and must be adjusted upward. This can be done as follows. Recall that the OTG algorithm aims at sampling options features that yield indifferent choices (on average). Therefore, within each cost bin, the target choice rate is 50%. Let *δ = 0.5-CR* be the difference between the choice rate *CR* within the selected cost bin and the target choice rate (i.e. 50% for indifference trials). We modify the previous small reward sample as follows:

1. $\left\{ \begin{aligned} r r+\left( r_{max}-r \right) \cdot T\cdot\delta if \delta<0 \\ r r+\left( r-r_{min} \right)\cdot T\cdot\delta if \delta>0 \end{aligned} \right.$

where $r_{min}$ and $r_{max}$ are the lower and upper bounds of the reward sampling grid, respectively (they correspond to 0.10€ and 29.90€ in this study), and $0<T<1$ is a weight that increases with the number of choices *N* previously made within the selected cost bin:

1. $T= \frac{2}{1+exp(2-N)}$

This renders the reward modification more stringent when the cost bin has been sampled more frequently over previous trials.

**Supplementary references**

1. J. Daunizeau, The variational Laplace approach to approximate Bayesian inference. *arXiv* (2017), doi:10.48550/arxiv.1703.02089.

2. J. Daunizeau, Semi-analytical approximations to statistical moments of sigmoid and softmax mappings of normal variables. *arXiv* (2017), doi:10.48550/arxiv.1703.00091.
